# Solid-phase XRN1 reactions for RNA cleavage: application in single-molecule sequencing

**DOI:** 10.1093/nar/gkab001

**Published:** 2021-01-28

**Authors:** Uditha S Athapattu, Charuni A Amarasekara, Jacob R Immel, Steven Bloom, Francis Barany, Aaron C Nagel, Steven A Soper

**Affiliations:** Department of Chemistry, University of Kansas, Lawrence, KS 66045, USA; Department of Chemistry, University of Kansas, Lawrence, KS 66045, USA; Department of Medicinal Chemistry, University of Kansas, Lawrence, KS 66045, USA; Department of Medicinal Chemistry, University of Kansas, Lawrence, KS 66045, USA; Weill Cornell Medical College, New York, NY 10065, USA; Sunflower Genomics, Inc., Lawrence, KS 66047, USA; Department of Chemistry, University of Kansas, Lawrence, KS 66045, USA; Sunflower Genomics, Inc., Lawrence, KS 66047, USA; Department of Mechanical Engineering and Bioengineering, University of Kansas, Lawrence, KS 66045, USA; Department of Cancer Biology and KU Cancer Center, University of Kansas Medical Center, Kansas City, KS 66160, USA

## Abstract

Modifications in RNA are numerous (∼170) and in higher numbers compared to DNA (∼5) making the ability to sequence an RNA molecule to identify these modifications highly tenuous using next generation sequencing (NGS). The ability to immobilize an exoribonuclease enzyme, such as XRN1, to a solid support while maintaining its activity and capability to cleave both the canonical and modified ribonucleotides from an intact RNA molecule can be a viable approach for single-molecule RNA sequencing. In this study, we report an enzymatic reactor consisting of covalently attached XRN1 to a solid support as the groundwork for a novel RNA exosequencing technique. The covalent attachment of XRN1 to a plastic solid support was achieved using EDC/NHS coupling chemistry. Studies showed that the solid-phase digestion efficiency of model RNAs was 87.6 ± 2.8%, while the XRN1 solution-phase digestion for the same model was 78.3 ± 4.4%. The ability of immobilized XRN1 to digest methylated RNA containing m6A and m5C ribonucleotides was also demonstrated. The processivity and clipping rate of immobilized XRN1 secured using single-molecule fluorescence measurements of a single RNA transcript demonstrated a clipping rate of 26 ± 5 nt s^−1^ and a processivity of >10.5 kb at 25°C.

## INTRODUCTION

With the development of next generation sequencing (NGS), the field of transcriptomics has seen tremendous advancements creating opportunities for improved diagnostics, prognostics, and treatment of diseases such as cancers and infectious diseases (1,2). RNA sequencing enables measurement of single nucleotide variants (SNVs), insertions and deletions, detection of different transcript isoforms, splice variants and chimeric gene fusions (1). There is also an increasing interest in the study of post-transcriptional modifications of RNA and their potential role in modulating processes associated with cancer and other diseases (3–6). Although NGS has been a useful technique for identifying specific post-transcriptional modifications, several technical challenges remain (7,8). Almost all current NGS techniques require library preparation prior to sequencing. During library preparation, the RNA molecules are fragmented and converted to cDNAs using reverse transcription and amplified using PCR, followed by a purification step (7). Not only does PCR introduce biases and other artifacts that would affect the identification and quantification of transcripts, but also by using these pre-sequencing steps important RNA modification information can be lost (9,10).

Single-molecule nanopore sequencing has been viewed as an attractive alternative to NGS that can address many of the aforementioned issues associated with NGS (11,12). Of the many potential advantages of single-molecule nanopore sequencing, the most exciting are the simple and inexpensive sample preparation steps, which do not in some cases require amplification using PCR, and in most cases provide longer reads compared to NGS. Unlike NGS, nanopore sequencing does not require fluorescent labelling as the sequencing is done using DNAs and RNAs in their native state, significantly reducing the sequencing cost and time (11). In addition, the lack of the need for amplification can preserve the post-transcriptionally modified ribonucleotides by not only eliminating PCR, but the reverse transcription step as well.

Nanopore sequencing is currently performed using two approaches, strand sequencing (13,14) or exosequencing (15). Although both methods have been used to sequence DNA, lesser considerations have been given to nanopore RNA sequencing. However, several reports do discuss RNA sequencing using both nanopore approaches (10,16–18). In one report, an engineered alpha hemolysin nanopore containing amino-cyclodextrin adapters were used together with an exoribonuclease enzyme, polynucleotide phosphorylase (PNPase), which cleaves single stranded RNA (ssRNA) in the 3′ → 5′ direction to produce ribonucleotide diphosphates (rNDPs). It was shown that the four canonical rNDPs could be discriminated using this exosequencing method with the additional charge on the rNDPs assisting in the capture of the cleaved rNDP by the nanopore (18). In another approach, strand sequencing of RNA was demonstrated using immobilized RNA, where the four canonical bases (adenosine, uridine, guanine, cytosine) and modified bases (I, m6A, m5C) were successfully distinguished (17). Exosequencing, where the biopolymer is cleaved into its constituent nucleotides in a sequential manner (either 5′ → 3′ or 3′ → 5′ direction) before passing through the nanopore is advantageous compared to strand sequencing because only one nucleotide is resident within the pore at any time (11). Thus, the resultant current transient signal resulting from a single nucleotide resident within the pore gives a distinguishable signal (11).

In this report, we lay the groundwork for an exosequencing technique for RNA using solid-state in-plane nanopores fabricated in thermoplastics, with exoribonuclease-1 (XRN1) immobilized onto a solid support (19–22). XRN1 is a processive exoribonuclease that cleaves ssRNA in the 5′ → 3′ direction releasing ribonucleotide monophosphates (rNMPs). XRN1 plays a critical role in RNA turnover and participates in nonsense-mediated decay, gene silencing, rRNA maturation, and degradation of mRNAs within eukaryotic cells (23–25). According to crystallographic data, the size of XRN1 is 15 nm × 15 nm × 15.49 nm at angles α = β = γ = 90° (23). The narrow entrance to the active site of XRN1, which is ∼9 Å, only allows the entry of 5′ monophosphorylated ssRNA and also helps in removing secondary structures as it cleaves through the substrate (23–25). The 5′ monophosphorylated ssRNA is required to be at least four nucleotides in length for efficient capture by the active site and the divalent cation Mg^2+^ acts as a cofactor to carry out its function as an exoribonuclease (25). The clipping rate of XRN1 *in vivo* has been reported to be 38–55 nt s^−1^ (26). Recently, Langeberg *et al.* measured the clipping rate of XRN1 *in vitro* and it was found to be 17.3 ± 0.6 nt s^−1^ at 37°C and pH 7.9 (27). However, to the best of our knowledge, there has been no published report on XRN1’s clipping rate and processivity when the enzyme is immobilized to a solid support. Furthermore, the presence of 45 lysine residues on XRN1 provides an abundance of potential attachment sites for covalent attachment onto a solid support bearing carboxylic acid groups using 3-(3-dimethylaminopropyl) carbodiimide/*N*-hydroxysuccinimide (EDC/NHS) coupling chemistry.

To understand the immobilization of XRN1 and its ability to cleave ssRNA into its constituent mononucleotides, we immobilized XRN1 onto pillars poised within a microfluidic device. Microfluidic devices, where enzymes are immobilized for biological reactions, are known as immobilized microfluidic enzymatic reactors, IMERs (28,29). There are several advantages of IMERs compared to solution phase bioreactors, such as enhanced enzymatic activity and stability, prevention of aggregation and auto-digestion, and reduced interference in downstream analysis (30,31). Previously, our group demonstrated that lambda exonuclease (λ-Exo), which cleaves double stranded DNA (dsDNA) to produce mononucleotides, can be covalently attached to a solid surface (20). Immobilized λ-Exo demonstrated an average clipping rate of 1100 ± 100 nucleotides per second (nt s^−1^), and a significantly higher processivity (∼40 000 bp) compared to the free solution enzyme.

Of the many substrates that are available (silicon, glass, polymers) for the fabrication of both microfluidic and nanofluidic devices, thermoplastics offer many advantages due to their favorable biocompatibility, good optical properties, ease of surface modification, and the number of well-established fabrication technologies to produce devices (32). The most commonly used thermoplastics for microfluidics are polycarbonate (PC), poly(methyl methacrylate) (PMMA) and cyclic olefin copolymer (COC) (20,32–34). In this study, we used PMMA as the substrate due to its favorable properties, such as good UV/vis transparency, low autofluorescence, and good solvent and acid/base resistance as well as its ability to be UV/O_3_ activated to generate surface confined carboxylic acid groups that can be used to attach biologics containing primary amine groups (35,36).

In this study, we report an IMER containing XRN1 as the immobilized enzyme for the sequential digestion of 5′ monophosphorylated ssRNA for potential applications in single-molecule RNA exosequencing. XRN1 was immobilized onto a UV/O_3_ activated PMMA device containing micropillars. Attachment consisted of using EDC/NHS coupling chemistry. AFM analysis showed that XRN1 only attached to the PMMA surface where it had been UV/O_3_ activated and in the presence of EDC/NHS with little or no nonspecific binding. Fluorescence studies, UPLC/MS measurements, and electrophoresis data provided information on the digestion of both modified and unmodified 5′ monophosphorylated RNA by both free solution and immobilized XRN1. Real-time digestion of dye labeled RNA by free solution and immobilized XRN1 was observed using fluorometry and fluorescence microscopy, respectively, allowing deduction of the processivity and clipping rate of both free solution and immobilized XRN1.

## MATERIALS AND METHODS

### Device fabrication and assembly

The IMERs used for these experiments were fabricated in PMMA (Plaskolite, Columbus, OH, USA) using hot embossing. The IMER contained a single channel that was 24 mm long and 1.4 mm wide. This channel contained 3600 micropillars with each pillar being 100 μm in diameter and 60 μm in height. The surface area and volume of this device were 1.22 cm^2^ and 2.9 μl, respectively (37). The device used for the real time monitoring of the immobilized enzyme's clipping rate and processivity using single-molecule fluorescence microscopy consisted of a single channel with no pillars that was 100 μm wide and 30 μm deep. A detailed procedure and schematic (see Supplementary Figure S1) for device fabrication and assembly can be found in the SI.

### Enzyme immobilization

**Figure 1.**
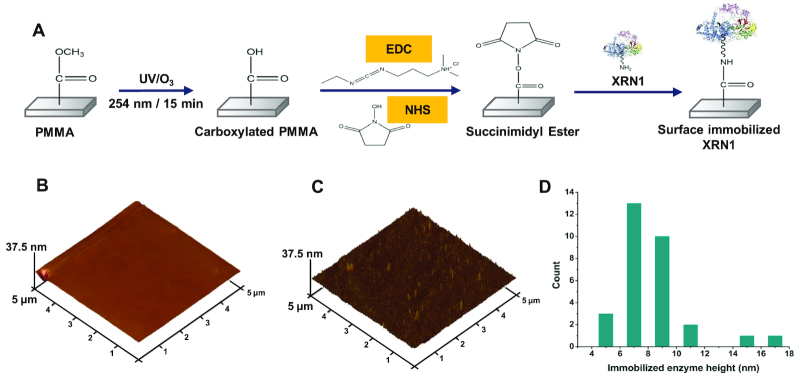
Covalent attachment of XRN1 onto UV/O_3_ activated PMMA. (**A**) Schematic representation of the process of covalent attachment of XRN1 onto PMMA surface by EDC/NHS coupling reaction. 5 μm × 5 μm AFM image of PMMA surface after UV/O_3_ activation, and incubation with 40 nM XRN1 enzyme (**B**) without (**C**) with EDC/NHS coupling reagents. (**D**) Height distribution of surface features present on (C). The average height of a surface structure is 8.4 ± 0.5 nm.

After fabrication and assembly of the microfluidic devices, XRN1 (NEB, Ipswich, MA, USA) was covalently immobilized onto microfluidic device surfaces using EDC/NHS coupling chemistry, which is used for attachment of primary amine containing biological entities, such as XRN1, to UV/O_3_ modified thermoplastic surfaces (36,38). A schematic representation of enzyme immobilization is shown in Figure 1A. The experimental procedure for enzyme immobilization is explained in detail in the SI.

### AFM analysis

To determine successful covalent attachment of XRN1 to activated PMMA surfaces, AFM analysis (Nanoscope IIIA, Brucker, MA, USA) was conducted. The tip used for imaging was a DLC-300 tip with a frequency of 300 kHz and a tip radius <15 nm. Tapping mode was used with a scanning frequency of 1 Hz so that possible damage done by the tapping force applied by the tip to the immobilized enzyme was minimal. PMMA surfaces (1 cm x 1 cm) were irradiated with UV/O_3_ light followed by the addition of a 40 nM XRN1 solution in the presence and absence (negative control) of EDC/NHS coupling reagents. The PMMA surfaces were kept at 4°C overnight and were rinsed with reaction buffer and distilled water and gently air dried prior to AFM imaging.

### Protein quantification

Pierce™ 660 nm protein quantification assay (Thermo Fisher Scientific, Waltham, MA, USA) was used to determine the amount of XRN1 covalently attached to the microfluidic channel containing micropillars. Absorbance of XRN1 solutions at 660 nm were measured (UV-VIS 1200 spectrophotometer, Shimadzu, Kyoto, Japan) before and after introduction into the IMER with attached enzyme. The calibration plot and a schematic of the experimental procedure used for protein quantification can be found in Supplementary Figure S2 in the SI.

### Digestion of monophosphorylated RNA

The model RNAs (60 nucleotides, nt) for XRN1 digestion studies were obtained from Integrated DNA Technologies, Inc. (Skokie, IL, USA). Following synthetic preparation, the 60 nt RNAs were purified using RP-HPLC and purity checked by mass spectrometry, which yielded a purity of 85–90%. The impurities were suspected to consist of truncated 60 nt RNAs lacking the 5′-monophosphorylated end and thus, would not serve as a viable substrate for XRN1 digestion.

Digestion of 5′ monophosphorylated 60 nt RNA was investigated using 2.32 pmol of XRN1 enzyme in both free solution and the immobilized state. In the free solution reaction, EDTA was added to stop the reaction after the desired time. The experimental control for the free solution reaction consisted of adding XRN1 to an RNA solution in the absence of the cofactor Mg^2+^. In the immobilized state, the effective reaction times were achieved by hydrodynamically pumping RNA solutions through the IMERs with a suitable flow rate using a syringe pump (Harvard Apparatus 22, Harvard Apparatus, Holliston, MA, USA). The negative control for the immobilized XRN1 reaction consisted of introducing RNA solutions to the IMERs, which did not contain immobilized XRN1. The pH was set at 7.9 for both solution-phase and solid-phase reactions. For all the XRN1 digestion experiments, ≥3 trials were conducted for each data point and the average value with the standard deviation is reported.

### Fluorescence measurements of 5′ monophosphorylated RNA

Following the XRN1 digestion, the remaining RNA molecules were labeled with SYTO 82 (Life Technologies, Eugene, OR, USA) to assess the extent of digestion. SYTO type dyes show a quantum efficiency of ∼0.4 when bound to RNA and a low quantum efficiency (0.01) in the presence of mononucleotides and the buffer alone (39). The fluorescence emission spectra of labeled RNA solutions were measured from 490 to 700 nm using a Fluorolog-3 fluorimeter (Horiba Jobin Yvon, Kyoto, Japan) with 480 nm excitation. The data was analyzed using Datamax 2.0 software.

### Denaturing microchip gel electrophoresis

The sizes of each ssRNA both before and after digestion by XRN1 were measured using denaturing microchip gel electrophoresis (Agilent Tapestation 2200 instrument; Agilent Technologies, Santa Clara, CA, USA). For the experiments reported herein, we used the high sensitivity RNA Screentape gel, which is a non-rigid plastic device that contains 16 lanes each of which are 25 mm in length, 2 mm in width and 1 mm in height. The denaturing gel (50–75% DMSO) consisted of 3% *N-*acryloylamido ethoxyethanol (AAEE). Gel electrophoresis data were analyzed using the Tapestation data analysis software. More details on the figures-of-merit of the denaturing microchip gel electrophoresis can be found in the SI.

### Analysis of digestion products by ultra-high-performance liquid chromatography (UPLC)/mass spectrometry (MS)

An analysis of the reaction mixture following the digestion of both unmethylated and methylated 60 nt RNA substrates with XRN1 was conducted using UPLC (Waters Acquity) coupled to a mass spectrometer (Advion Expression^s^ CMS mass spectrometry-electrospray ionization system). The experimental procedure is provided in detail in the SI.

### *In vitro* transcription (IVT) of full-length firefly luciferase (FLuc) and human Duchenne muscular dystrophy gene (DMD) RNA transcripts

For real time RNA digestion studies, 1766 nt (FLuc) and 11 163 nt (DMD), homogeneous ssRNA molecules were synthesized using IVT (see Supplementary Figure S3A and S3B in SI). A detailed explanation of IVT can be found in the SI.

### 5′ Monophosphorylation of RNA

Following IVT, the resultant 5′ triphosphorylated ssRNA was treated with RNA 5' Pyrophosphohydrolase (RppH) to produce 5′ monophosphorylated RNA, which served as a substrate for XRN1 (Supplementary Figure S3 in SI). Additionally, to demonstrate the removal of the 5′ cap structure of the RNA to generate a viable substrate for XRN1, we used IVT 62mer RNA and CleanCap Fluc RNA (TriLink Bio Technologies, San Diego, CA, USA) both containing a cap1 structure at their 5′ ends. The IVT 62mer RNA was capped using ‘one-step capping and 2′-*O*-methylation protocol’ that generates a cap1 structure at the 5′ end of the RNA following the manufacturer's recommended protocol (NEB, Ipswich, MA, USA). The capped RNAs were treated with mRNA decapping enzyme (MDE) to remove the 5′ cap structure for subsequent digestion by XRN1 (Supplementary Figure S4 in SI). More details of RNA capping and decapping can be found in the SI.

### Clipping rate and processivity of XRN1

For determination of the clipping rate and processivity of XRN1 in free solution, a method described by Han *et al.* was used with slight modifications (40). First, 0.0875 pmol of RiboGreen dye (Life technologies, Eugene, OR, USA) labelled FLuc RNA was incubated with 0.35 pmol of XRN1 in the presence of XRN1 buffer (100 mM NaCl, 50 mM Tris–HCl, 1 mM DTT) without Mg^2+^ to enable complexation of FLuc RNA to XRN1 without clipping. Next, 8.75 pmol of a competitor RNA, in this case a 60 nt RNA saturated with SYTO 82 dye (Life Technologies, Eugene, OR, USA) in XRN1 buffer with Mg^2+^ (final Mg^2+^ concentration of 20 mM) was added to FLuc RNA complexed to XRN1, and the fluorescence intensity of the mixture was measured at 30 s intervals for 30 min with 470 nm excitation and 500 nm emission using the Fluorolog-3 fluorimeter. Data were analyzed using Datamax 2.0 software.

To deduce the clipping rate and processivity of immobilized XRN1, a method described by Oliver-Calixte *et al.* was used (20). XRN1 was immobilized to the cover plate of the single channel microfluidic device (no pillars) and SYTO 82 labeled DMD RNA was introduced into the device in enzyme buffer without Mg^2+^ to facilitate complexation of DMD RNA with immobilized XRN1. Complexed DMD RNA to XRN1 was determined by monitoring the fluorescence of single RNA molecules to make sure that they were not randomly diffusing (see SI for more details). Once a single DMD RNA molecule was located that was complexed to the immobilized XRN1, enzyme buffer containing Mg^2+^ cofactor was introduced into the device to initiate digestion and the fluorescence intensity (532 nm excitation, 0.01 W) of the DMD RNA–XRN1 complex was monitored continuously. For these experiments, an epifluorescence microscope was used, which consisted of a NIKON TE 2000 microscope fitted with a 100X/1.4 NA oil-immersion objective and an Andor iXon3 EMCCD camera (21). All images were acquired using Metamorph advanced v7.5.6.0 software (10 fps acquisition rate). Acquired images were analyzed using Image J software.

### Statistical analysis

All reported data sets were compared by a two-sided t-test using R Studio v1.0.153 and R v3.5.1 software.

## RESULTS

### XRN1 immobilization and quantification

XRN1 contains a total of 45 lysine residues, most of which reside opposite to the active site. These lysine residues act as attachment sites available for conjugation to –COOH functional groups on the UV/O_3_ activated PMMA surface. To confirm successful covalent attachment of XRN1 onto UV/O_3_ activated PMMA surfaces using EDC/NHS coupling chemistry (see Figure 1A), an AFM analysis was carried out to determine the presence of morphological features indicative of covalently immobilized XRN1. Sheet PMMA surfaces were exposed to UV/O_3_ light and a 40 nM XRN1 solution was introduced without and with EDC/NHS reagents (Figure 1B and C, respectively). As shown in Figure 1B and 1C, surface features could be seen in EDC/NHS treated PMMA surfaces with the absence of such features in the case of no EDC/NHS reagents.

To determine the heights of the surface features present in EDC/NHS treated PMMA surfaces, surface structures in Figure 1C were measured (see Figure 1D). According to the height distribution, the average height was determined to be 8.4 ± 0.5 nm, which was near the size of this molecule in terms of its crystal structure, which is ∼15 nm (23). The slight disparity in size could be due to the compression of the enzyme by the tapping force applied by the AFM tip and/or size reduction of the enzyme due to dehydration (41,42).

We used a protein quantification assay to determine the amount of XRN1 covalently attached inside the micropillared IMER (surface area 1.17 cm^2^). For these experiments, three different input concentrations of XRN1 were used (183, 305, 426 nM) based on the amount of XRN1 needed for a theoretical monolayer coverage of the IMER (1.1 × 10^11^ molecules), and the lowest XRN1 enzyme concentration that can be measured using the protein quantification assay. The extent of nonspecific adsorption was assessed by introducing XRN1 solutions to IMERs that had not been treated with EDC/NHS coupling reagents following UV/O_3_ activation of the polymer.

In the absence of the coupling reagents, XRN1 would only adsorb to the surface non-specifically and the number of moles was calculated to be <2% of the input number of moles for each XRN1 concentration (see Supplementary Table S1 in SI). When the IMERs were treated with EDC/NHS coupling reagents for 15 min prior to flowing XRN1 solutions through the devices, the number of moles of enzyme covalently attached increased with increasing input XRN1 concentration and ranged from 2.32 to 4.07 pmol of XRN1 (See Supplementary Table S2 in SI). The total number of moles immobilized from the total input number of moles ranged from 53.4% to 39.4% while the surface density of immobilized enzyme ranged from 1.98 to 3.48 pmol/ cm^2^.

### Digestion studies of 5′ monophosphorylated RNA

**Figure 2.**
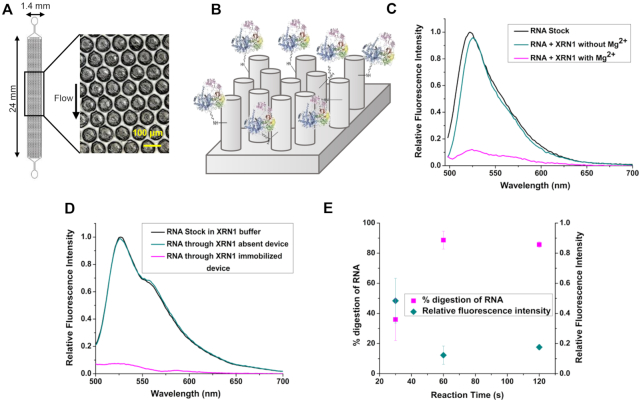
Solid-phase digestion reactions of XRN1. (**A**) Top down view of the pillared IMER channel. (**B**) Schematic representation of the covalently attached enzyme on the micropillars of the device. Fluorescence emission spectra of SYTO RNASelect Green labeled monophosphorylated RNA solutions digested by XRN1 in (**C**) free solution and (**D**) Immobilized state. The reaction time was 60 s and 2.32 pmol of enzyme was used in both free solution and immobilized digestion. SYTO RNASelect Green was added after digestion and fluorescence emission spectra were taken from 495 to 700 nm with 480 nm excitation. (**E**) Percentage digestion and relative fluorescence intensity of digested RNA with varied reaction time and constant surface enzyme density. The XRN1 reactions were all performed at room temperature. The error bars represent standard deviations in the measurements (*n* ≥ 3).

To demonstrate the ability of XRN1 to digest monophosphorylated RNA, 10.6 pmol of a 60 nt RNA substrate was reacted with 2.32 pmol of XRN1 both in solution- and the solid-phase. For solid-phase XRN1 experiments, we used a micropillared IMER consisting of 3600 micropillars (see Figure 2A). A schematic representation of the enzyme-immobilized IMER is shown in Figure 2B. Fluorescence emission spectra shown in Figure 2C depict the digestion of RNA by free solution XRN1 in the presence and in the absence of the cofactor, Mg^2+^. As can be seen from the spectrum depicted in dark cyan in Figure 2C, in the absence of Mg^2^^+^ with XRN1 present there was no change in the fluorescence spectrum of the RNA labeled with SYTO RNASelect Green compared to the RNA solution with no XRN1, indicating the 60 nt RNA remained intact after 60 s. When the Mg^2+^ cofactor was introduced into the reaction mixture, the 60 nt RNA was digested as shown by the loss of fluorescence due to cleavage of the RNA. Peak area analysis of the spectra yielded a digestion efficiency of 78.3 ± 4.4% (*n* = 3; *T* = 25°C) after 60 s for XRN1 solution-phase reactions.

Figure 2D shows fluorescence spectra of SYTO RNASelect Green-labeled 60 nt RNA reacted with XRN1 when immobilized within the IMER. The negative control for this experiment consisted of flowing 10.6 pmol of the 60 nt RNA substrate through the IMER that did not contain immobilized XRN1. The negative control revealed that there was no loss of intact RNA molecules as evident by the emission spectrum appearing similar to the RNA stock solution. When the 60 nt RNA was flowed through the IMERs containing immobilized XRN1, the amount of intact RNA decreased as apparent from the loss of fluorescence seen in the magenta trace in Figure 2D. Peak area analysis of the spectra indicated that 87.6 ± 2.8% (*n* = 4; *T* = 25°C) of the 60 nt RNA was digested by the immobilized XRN1 enzyme. However, these numbers should be qualified by the fact that the RP-HPLC purified 60 nt models contained RNA fragments that were not 5′-monophosphorylated making them a non-viable substrate for XRN1.

To assess the effect of surface enzyme density on the activity of immobilized XRN1, the 60 nt RNA substrate was introduced into the XRN1 immobilized IMERs for 60 s with different XRN1 concentrations used for the immobilization reaction. The digestion percentages were >80% for all surface enzyme densities used (Table 1) and showed no significant statistical difference at the 95% confidence level as determined by a *t*-test.

**Table 1.** Percent digestion as a function of enzyme load

Varied enzyme concentration			
**pmol of enzyme**	**2.32 (*n* = 3)**	**2.97 (*n* = 3)**	**4.07 (*n* = 3)**
**% RNA digestion**	88.7 ± 5.9	83.8 ± 6.9	82.9 ± 8.7

Next, to evaluate the effect of reaction time on the percent digestion of the 60 nt RNA substrate, surface enzyme density and the concentration of RNA solutions were kept constant, while the reaction times were varied by changing the flowrate of the RNA substrate through the IMERs. When a reaction time of 30 s was used, the digestion percentage was ∼35% and when the reaction time was increased to 60 and 120 s, the digestion percentages were >80% (see Figure 2E) and showed no significant difference at the 95% confidence level (*P* = 0.5284).

### Ability of XRN1 to digest through methylated RNA sequences

**Figure 3.**
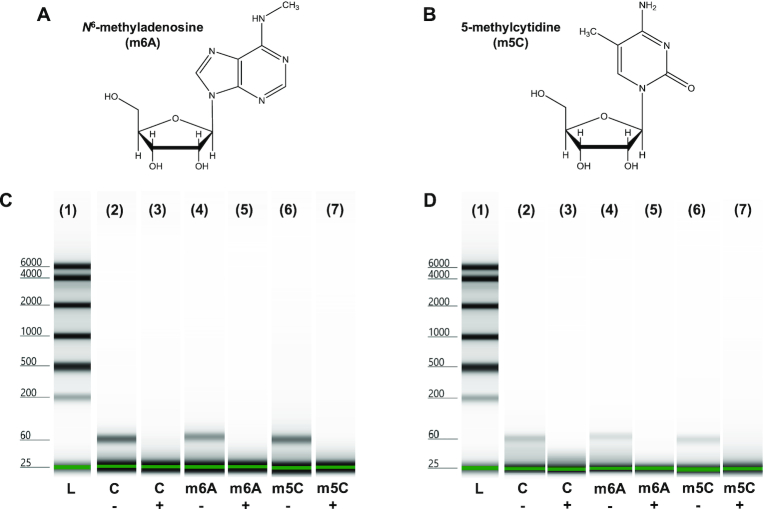
Digestion of methylated RNA sequences. Chemical structures of (**A**) m6A and (**B**) m5C. Digestion of methylated RNA sequences by (**C**) solution phase and (**D**) immobilized XRN1. (1) Ladder (L). negative control for (2) unmethylated (c–) (4) m6A-methylated (m6A–) and (6) m5C-methylated (m5C–) RNA. Digestion results for (3) unmethylated (c+) (5) m6A-methylated (m6A+) and (7) m5C-methylated (m5C+) RNA by XRN1.

We next investigated the ability of both solution-phase and solid-phase XRN1 to digest through sequences that contained methylated bases using two 60 nt RNA sequences. Each 60mer contained one of the two most common RNA modifications found in eukaryotic cells. One RNA sequence contained an *N*^6^-methyladenosine (m6A) residue (see Figure 3A) at the fifth nucleotide position within the 60mer, while the second RNA sequence contained a 5-methylcytosine (m5C) residue (see Figure 3B) at the 10th nucleotide position from the 5′ end. A third unmethylated RNA sequence was used as the control sequence. The sequence of each RNA 60mer is shown in Supplementary Figure S5A–C in the SI.

Each RNA sequence was reacted with immobilized XRN1 in the IMER for 60 s. RNA in the absence of XRN1 were used as the negative control. After the reactions were complete, denaturing microchip gel electrophoresis was conducted to determine the length of the remaining RNAs. If XRN1 was unable to digest through the methylated nucleotides, RNAs with a length of 51 nucleotides should remain for the m5C RNA and 56 nucleotides for the m6A RNA. As can be seen from Figures 3C and D, peaks corresponding to 60, 51 or 56 nucleotides were not observed after 60 s of reaction for both solution and IMER reactions demonstrating the ability of XRN1 to digest through RNA structures containing m6A and m5C residues. For more information on the electrophoresis figures-of-merit of the Tapestation high sensitivity RNA Screentape, please see Supplementary Figure S5D in the SI. We note that the signal intensity difference in the negative controls between solution-phase and the solid-phase XRN1 reactions is likely due to some sample loss while collecting the sample eluent from the IMERs.

To determine the extent of digestion of methylated RNAs by the immobilized XRN1, the IMER digested RNA solutions were stained with SYTO 82 post-digestion and the fluorescence emission spectra were taken from 490 to 700 nm with 480 nm excitation. Peak area analysis of these spectra revealed that after 60 s of reaction, 87.0 ± 4.2% (*n* = 4; *T* = 25°C) of m6A methylated RNA was digested (see Supplementary Figure S5E), while after the same amount of time, 77.3 ± 6.0% (*n* = 3; *T* = 25°C) of m5C methylated RNA was digested by the immobilized XRN1 (see Supplementary Figure S5F). The digestion of m5C RNA seemed to be somewhat slower compared to the m6A RNA for the surface immobilized XRN1 (*P* = 0.0243). If XRN1 digestion was terminated at the methylation sites, the fluorescence intensity would be closer to that of the negative control due to the fact that the oligomers remaining (56 nt and 51 nt) were close in size to the starting RNA 60mer.

We also subjected both unmethylated and methylated RNA strands digested by XRN1 to UPLC/MS analysis. As a reference, we ran an rNMP standard mix containing each rNMP in the expected concentration if the input 60mer RNA was fully digested by XRN1 (see Supplementary Figure S6A). If XRN1 was unable to digest through the m6A methylated RNA, only the first four nucleotides would be cleaved by XRN1 resulting in an RNA product of 56 nt. Therefore, the four nucleotides, rAMP, rUMP, rCMP and rGMP, would appear in the chromatogram in a 1:2:1:0 intensity ratio, respectively. Moreover, m5C would generate a 2:5:1:1 (A:U:C:G) ratio if the digestion was terminated at the methylation site. These intensity ratios were not observed. The peaks for each ribonucleotide for XRN1 60mer RNA reactions were in the expected intensity ratio to a fully digested 60mer RNA (see Supplementary Figures S6B–D).

Furthermore, the UPLC/MS results indicated that the digestion products of XRN1 were indeed 5′ rNMPs and the methylations in the resultant rNMPs were preserved (see Supplementary Figure S6E). We investigated the mass spectra of both the rNMP mixture and m6A methylated RNA to determine the composition of the overlapped UPLC peaks at 4.2 and 4.4 min, which could have arisen from rGMP and m6-rAMP or 8-oxo-guanosine monophosphate. Guanosine is the most susceptible nucleotide to oxidation with an oxidation product 8-oxo-guanosine monophosphate, which has a molecular weight of 379.2 g/ mol (43). The [M+H] mass spectrum for either the ribonucleotide mixture or the m6A–RNA did not contain a peak at 380.2, which indicated that 8-oxo-guanosine was not found.

### Clipping rate and processivity of XRN1

The clipping rate and processivity of XRN1 are important parameters in understanding the enzyme activity both in free solution and the immobilized state for a number of applications. Thus, we assessed these properties of both the free solution and immobilized XRN1 using IVT RNA substrates.

**Figure 4.**
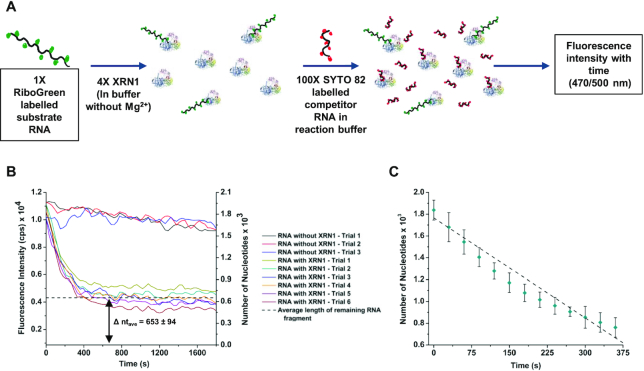
Solution phase clipping rate and processivity of XRN1. (**A**) Schematic representation of the reaction procedure. (**B**) Fluorescence intensity of RiboGreen labelled FLuc RNA with time. According to the average length of FLuc RNA fragment remaining after the reaction (Δ nt_ave_), the processivity of XRN1 in solution phase is 1113 ± 132 nucleotides. (**C**) Clipping rate calculated using the fluorescence decay portion from 5A. According to the slope of the graph (*R*^2^ = 0.99121), the average clipping rate of XRN1 in solution is 3.06 ± 0.11 nt s^–1^ at 25°C.

To determine the clipping rate and processivity of free solution XRN1, we used FLuc RNA labelled to saturation with RiboGreen dye as the substrate and a 60 nt RNA as the competitor that was labeled with SYTO 82 (see Figure 4A). Experiments carried out using RiboGreen labelled and unlabeled FLuc RNA showed that there was no statistical difference in digestion rates for the labeled vs. unlabeled substrates at the 95% confidence interval (*P* = 0.5196; see Supplementary Figure S7A in SI). To prevent the released RiboGreen dye molecules from attaching to the competitor 60mer RNA generating a fluorescence background, the competitor RNA molecules were labelled to saturation with SYTO 82, which did not produce a fluorescence signal using the RiboGreen filter set due to spectral dissimilarities between these dyes. If the processivity of XRN1 is below 1766 nucleotides, the FLuc RNA will detach from the enzyme and the re-engagement of the partially digested FLuc RNA to XRN1 will be prevented by the smaller competitor 60mer RNA, which was in a 100-fold molar excess compared to the FLuc RNA. Therefore, because undigested FLuc RNA will show fluorescence in the RiboGreen spectral range specifically, partial cleavage will give a constant fluorescence signal after the reaction was terminated due to the remaining residual FLuc RNA.

As shown in Figure 4B, the fluorescence intensity of the solution decreased with time and came to a constant value at ∼330 s. The background was measured in the presence of SYTO 82 labeled 60 nt RNA and RiboGreen dye in the absence of FLuc RNA. The background signal was subtracted from the fluorescence emission spectra shown in Figure 4B. Using a calibration plot between the fluorescence intensity and the number of nucleotides (see Supplementary Figure S7B in SI), the constant fluorescence value was converted to the number of nucleotides, which was found to be 653 ± 94 nt (Δnt_ave_). The Δnt_ave_ represented the average length of FLuc RNA remaining after the XRN1 reaction, which indicated that the processivity of XRN1 in free solution was 1113 ± 132 nt. Using the fluorescence decay portion of Figure 4B, the clipping rate of XRN1 was 3.06 ± 0.11 nt s^−1^ (*n* = 6, *R*^2^ = 0.99332) at 25°C (see Figure 4C).

For determining the clipping rate and processivity of immobilized XRN1, we monitored the fluorescence of a single DMD RNA (11.1 kb) molecule labeled with SYTO 82 and associated to a single immobilized XRN1 molecule using a high-sensitivity fluorescence microscope equipped with an EMCCD camera. In this case, we used a longer RNA strand compared to FLuc to produce a brighter fluorescence signal from a single RNA molecule. Analysis of the fluorescence emission obtained for DMD RNA labeled with SYTO 82 both pre-digestion and post-digestion did not show a statistical difference at the 95% confidence interval (*P* = 0.1573) indicating that the labeling had no influence on the activity of XRN1 (see Supplementary Figure S7C in SI).

**Figure 5.**
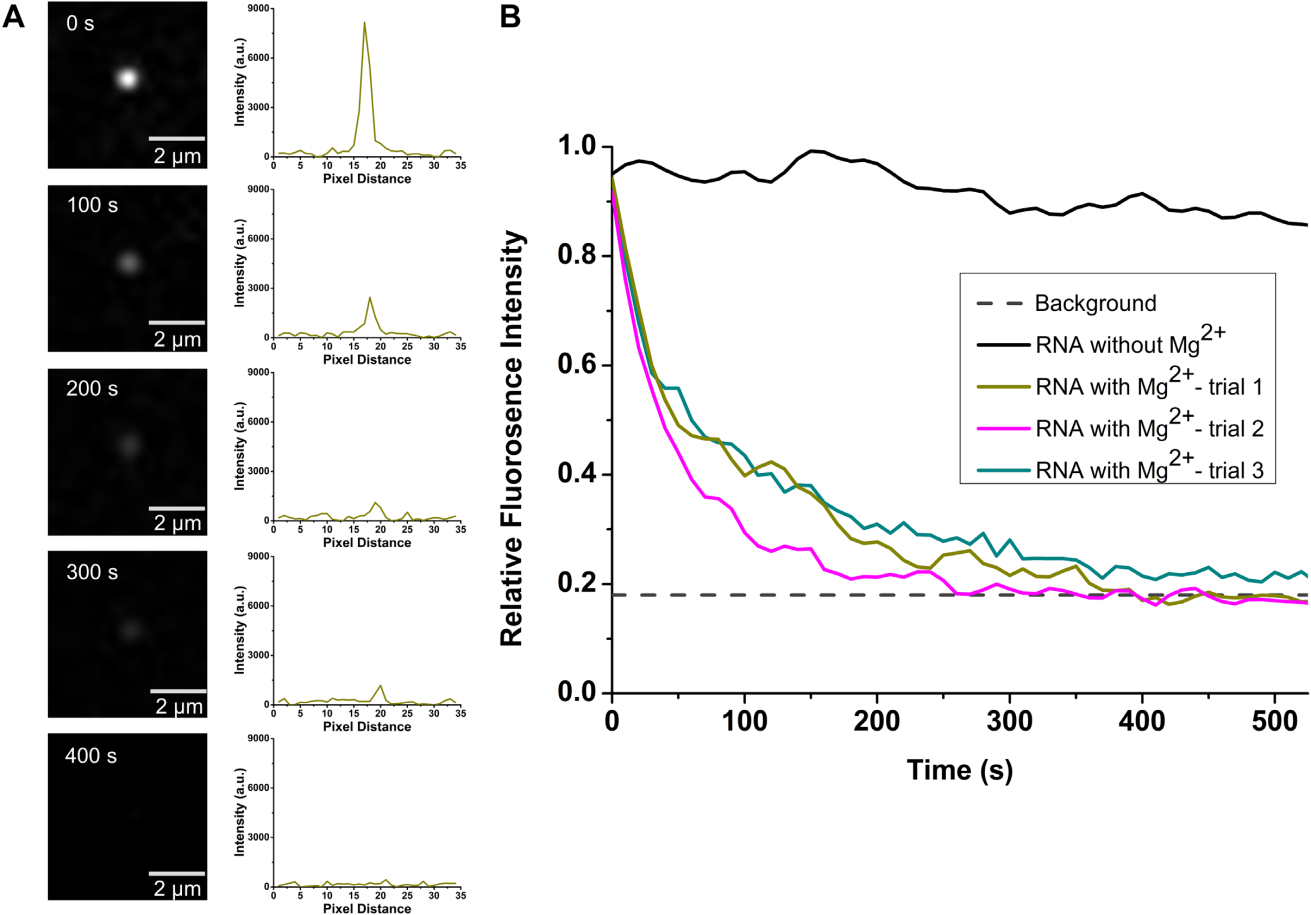
Digestion of SYTO 82 labeled DMD RNA by immobilized XRN1. (**A**) Fluorescence still images and corresponding intensity plot profiles of labeled DMD RNA-immobilized XRN1 complex acquired at different times, after introduction of Mg^2+^ to initiate digestion. (**B**) Relative fluorescence intensity of RNA–enzyme complexes with time. The black spectrum depicts the intensity of the complex in the absence of the cofactor Mg^2+^. The dark cyan, dark yellow and magenta spectra illustrate the fluorescence intensity of the complexes when Mg^2+^ is introduced. The average fluorescence intensity becomes indistinguishable from the background intensity ∼400 s.

XRN1 was immobilized onto the cover plate of a single-channel microfluidic device made from PMMA. Then, single DMD RNA molecules were flowed hydrodynamically through the microchannel and when the fluorescence generated from a single RNA molecule was found to remain stationary, it was assumed to be associated to the XRN1 immobilized enzyme (see Supplementary Figure S8A in SI). Unassociated RNA molecules moved in and out of the field-of-view of the microscope when the flow was stopped (see Supplementary Figure S8B in SI). The enzyme cofactor (Mg^2+^) was then flowed into the microchannel and the fluorescence was monitored in real time to determine the processivity and clipping rate. When buffer containing Mg^2+^ was introduced into the microfluidic device, the fluorescence intensity of the stationary RNA molecules decreased with time (see Figure 5A). To confirm that the fluorescence loss was due to clipping of RNA by XRN1 and not to photobleaching, control experiments were carried out in which the stationary RNA molecules were exposed to the excitation light and the fluorescence intensity was measured as a function of time. As can be seen in Figure 5B, there was no significant reduction of fluorescence intensity of RNA molecules when the Mg^2+^ cofactor was absent. This is also in agreement with the literature, which reported that SYTO 82 exhibits low levels of photobleaching (39). To determine the shortest detectable SYTO 82 labeled RNA fragment, a calibration plot was constructed using DMD RNA and FLuc RNA with SYTO 82 labeling (Supplementary Figure S7D in SI). According to the calibration plot, the smallest RNA fragment that could be detected was 664 nt.

During the fluorescence imaging experiment, a single RNA molecule associated with the immobilized XRN1 did not move out of the microscope's field-of-view, indicating that the RNA molecule was associated to the immobilized XRN1 and when the fluorescence spot being imaged disappeared, the remaining fragment of RNA, if present, was below 664 nt. As can be seen from Figure 5A, for a single RNA molecule, the signal completely disappeared. This suggested that the apparent processivity of immobilized XRN1 should be greater than or equal to 10 499 nt. We note that the processivity of XRN1 in the immobilized state is given as an apparent processivity due to the indirect nature of obtaining the data (20).

The average clipping rate of XRN1 in the immobilized state was deduced using the decay portion of the fluorescence intensity shown in Figure 5B. The total observable length of DMD RNA was calculated by subtracting the smallest detectable length from the length of DMD RNA. The clipping rate was then calculated by dividing the total observable length by the time the relative fluorescence intensity reached background (400 s). This yielded an average clipping rate of 26 ± 5 nt s^−1^ (*n* = 3) for immobilized XRN1 at 25°C. We note that the clipping rate and the processivity of both free solution and immobilized XRN1 may not be optimal values as these experiments were conducted at room temperature (25°C) instead of the enzyme's optimum temperature of 37°C.

## DISCUSSION

RNA sequencing has become extremely important due to the recent COVID-19 pandemic. Reports using Illumina-based NGS have determined that this coronavirus, called SARS-CoV-2, has an approximate 30 kb ssRNA genome with a sequence different from that of the 2002 SARS coronavirus (∼79% sequence homology) and the MERS virus (∼50% sequence homology) (44–46). Due to the evolutionary rate of RNA viruses (∼10^−4^ nt substitutions per year), RNA sequencing will continue to be an important tool for not only detection, but vaccine discovery and determining resistance as well. Thus, new strategies for RNA sequencing that can provide simpler workflow, longer reads, and amplification-free formats would be particularly attractive. We have previously reported a single-molecule DNA sequencing strategy that fits the aforementioned operational criteria (19–22). Briefly, it uses a processive exonuclease tethered to a solid-support with the cleaved nucleotides shuttled electrokinetically through a nanometer channel that measures the molecular-dependent electrophoretic mobility as an identifier. However, we are now envisioning this single-molecule sequencing strategy for RNA sequencing. As a foundation for that transition, we investigated the ability to use a solid-phase exoribonuclease reaction to sequentially generate ribonucleotides for identification using a label-free approach with high base identification accuracy via mobility matching and can identify modified ribonucleotides as well due to the lack of need for a PCR step in the workflow.

There are two categories of ribonucleases, endoribonucleases and exoribonucleases. Endoribonucleases cleave RNA internally whereas exoribonucleases cleave RNA sequentially from either the 3′ or 5′ end (47). Exoribonucleases are further categorized into two types, distributive enzymes in which the RNA substrate is separated from the enzyme after each catalytic event and, processive enzymes where the RNA substrate is held by the enzyme until all of the nucleotides are cleaved from the intact substrate or the enzyme decomposes (48). For identification of rNMPs or rNDPs using an exosequencing technique, sequential clipping of the nucleic acid substrate with high fidelity is critical (13,15,18,49).

The processivity is important in exosequencing as well because it is a factor that determines read length. Of the many exoribonucleases available only a few are processive (50). Among these are the 5′→3′ exoribonucleases (XRNs): XRN1 and XRN2 (Rat1) and 3′→5′ exoribonucleases RNase II, RNase R, and PNPase (23–25,50,51). The 3′→5′ exoribonucleases act on either 3′-OH or 3′-phosphate to produce rNMPs or rNDPs (52–54). Although the 3′→5′ exoribonucleases eliminate the need for prior sample treatment such as 5′-m^7^G decapping and dephosphorylation (55,56), both RNase II and RNase R leave a residual oligonucleotide that is 3–5 nucleotides in length (53). In the case of RNase II and PNPase, the enzyme activity is stalled by stable secondary structures in the RNA substrate (54).

XRNs digest 5′ monophosphorylated RNA (23–25,51,55,56) to produce rNMPs as has been shown for XRN1 (55). It has been reported that the activity of XRN2 is stalled when encountering secondary structure in the RNA substrate (51). XRN2 can cleave through RNA strands with stem-loop structures only in the presence of the Rai1 protein (51). Unlike XRN2, XRN1 can digest through RNA secondary structures due to the size of its active site and the mechanism of its action as demonstrated in several reports (23–25). However, the narrow active site of XRN1 (∼9 Å) does prevent access of double stranded RNA structures (23–25). The 5′ monophosphorylated ssRNA substrate is pulled through the narrow gap by a Brownian ratchet mechanism and this together with the steric barrier at the entrance causes duplex unwinding (23–25). In addition to being processive, the ability to digest through secondary structures is a major advantage of using XRN1 in an exosequencing method, as it will eliminate the need for prior sample preparation to remove secondary structures in the RNA substrate.

As noted in the SI, the model RNA substrates used in this study possessed significant secondary structures (see Supplementary Figure S9). For example, the 60 nt RNA model was exhaustively digested by both the solid-phase and solution-phase XRN1 reactions (see Figure 3), in spite of the fact that this 60 nt RNA model possessed a stable secondary structure starting at the fifth nucleotide from the 5′ end. If XRN1 was unable to cleave through this secondary structure, a 56-nucleotide fragment would have appeared in the gel trace shown in Figure 3 following the reaction and this was not the case.

**Figure 6.**
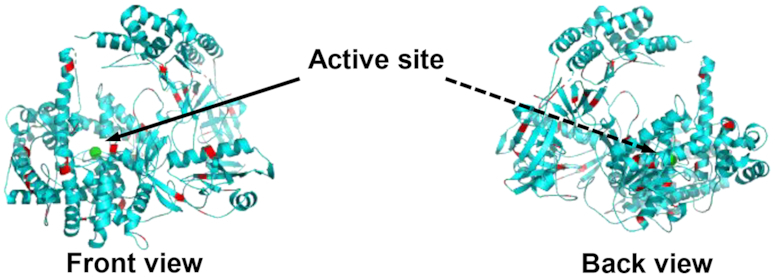
Front and back view of XRN1 with lysine groups highlighted in red. The lysine residues on the surface of the enzyme indicate potential attachment sites to PMMA surface. Structure of XRN1 was obtained from RCSB protein data bank and modified using PYMOL v2.1.1 software.

Moreover, the presence of 45 lysine groups on the surface of XRN1 as potential attachment sites onto a solid support possessing –COOH groups makes XRN1 an ideal exoribonuclease for exosequencing requiring a solid-phase reaction. For example, the UV/O_3_ activation of many plastics, such as PMMA, creates surface-confined carboxylic acid groups that can be coupled to primary amines found on lysine groups using EDC/NHS coupling chemistry (see Figure 1A). Figure 6 shows the primary structure of XRN1 with the lysine groups highlighted in red. Of the many sites available for covalent attachment to the carboxylated surface, only ∼4 lysine residues reside in close proximity to the active site while most of the reactive lysine residues are located on the opposite side of the active site, which allows the efficient capture of RNA substrates by the immobilized enzyme without masking of the active site by the surface.

As previously reported, the –COOH group density of UV/O_3_ activated polymers depend on the polymer type and the UV dose (38). For enzyme immobilization onto PMMA, a lower surface density of –COOH groups is desirable to avoid multisite attachment of the enzyme to the surface, which could inadvertently lead to an inaccessible active site or denaturation of the enzyme (57,58). Therefore, immobilization of XRN1 that minimizes multi-site attachment is critical for efficient accessibility to the active site and sequential digestion of an RNA substrate. The conditions used here for attachment of XRN1 onto PMMA (UV dose of 16.0 mW/ cm^2^ for 15 min) generates a –COOH group density of 32 pmol/ cm^2^ as determined by a TBO assay (20). This is ∼10 times higher than the highest surface concentration used here (see Table 1), which may result in multi-site attachment. However, the TBO assay, which was used to quantify the amount of -COOH groups on plastics, tends to label –COOH groups underneath the surface of the PMMA substrate that are not accessible by the enzyme (20).

Immobilization of enzymes can, however, cause conformational changes altering performance (59). Although in most cases these conformational changes lead to adverse effects in terms of enzyme activity, sometimes these conformational changes can lead to enhanced enzyme activity, stability and specificity (31). Immobilization can also increase the rigidity of the enzyme, which can result in higher stability (60,61). Previously we observed that immobilized λ-Exo, which is comprised of three subunits, demonstrated a processivity much higher than solution-phase reactions most likely arising from enzyme stabilization (20). Proper immobilization of the enzyme to the solid support will stabilize the enzyme and maintain its structure in turn increasing the enzyme processivity and even the clipping rate (31,60). It is also reported in some cases that increasing the rigidity of an enzyme can reduce allosteric inhibition leading to higher enzyme activity (62).

As noted from our data shown in Figures 4 and 5, the clipping rates of XRN1 were 3.06 ± 0.11 and 26 ± 5 nt s^−1^ at 25°C for solution-phase and solid-phase reactions, respectively. Langeberg *et al.* recently reported the free solution clipping rate of XRN1 was 17.3 ± 0.6 nt s^−1^ (27). This value was ∼6-fold higher compared to the value we obtained and is most likely due to differences in enzyme reaction temperatures (37°C versus 25°C). We should also note that solution-phase (Figure 4) and the solid-phase (Figure 5) clipping rates reported in this manuscript were secured using bulk and single-molecule measurements and different templates of RNA, which could have contributed to the observed differences. At any rate, because the immobilized XRN1 enzyme can function in a fashion similar to the solution enzymatic reaction supports our supposition that XRN1 can potentially be used for single-molecule exosequencing requiring an immobilized enzyme.

More than 170 post-transcriptional RNA modifications have been identified to date (63). Most of these modifications occur in abundant non-coding RNAs including ribosomal RNA (rRNA) and transfer RNA (tRNA) and play an important role in structural folding and function. There are six nucleotides with base modifications that can influence the metabolism and function of messenger RNA (mRNA): m6A, m5C, inosine (I), pseudouridine (ψ), *N^1^*-methyladenosine (m1A) and 5-hydroxylmethylcytidine (5-hmC). Additional functions of RNA modifications include tRNA stability, cellular stress response (m5C), and microRNA stability (2′-*O*-methlyation). The ‘epitranscriptome’, which is the term used to describe RNA modifications throughout the transcriptome (64), has historically been difficult to study due to a lack of tools for deciphering the presence of the entire inventory of RNA modifications. However, technological advancements such as NGS have led to a greater understanding of the epitranscriptome and its role in normal biology and disease (65). Recently, Koh *et al.* demonstrated the use of XRN1 to map exact locations of m6A modifications within an RNA molecule (66). This technique, coined m6A-crosslinking-exonuclease-sequencing (m6ACE-seq), involves anti-m6A antibodies photo-crosslinked onto m6A and 2′-*O*-methylated m6A (m6Am) modifications contained within an RNA molecule. The antibody crosslinking halts the digestion of RNA by XRN1 at the exact position of m6A/ m6Am thereby indicating the position of these methylations.

For the RNA nucleotide modifications we tested, we found that XRN1 could cleave through the m5C and m6A modified nucleotides. This is in agreement with the literature, which has reported that XRN1 is involved in degradation of m6A and m5C containing RNA species (67–71). Both m6A and m5C were chosen as RNA models for this initial work based on literature precedence indicating the high abundance of these modifications throughout the transcriptome, which has been corroborated using next-generation RNA-sequencing (72–74).

Our conclusion of XRN1’s ability to cleave through m5C and m6A nucleotides was supported by three lines of independent evidence: (i) Denaturing gel electrophoresis, which showed the disappearance of bands near the intact 60mer RNA model. (ii) Near complete loss of fluorescence specific to intact RNA molecules. (iii) UPLC/MS data obtained that indicated the molar ratios of the ribonucleotides was consistent with complete digestion with the structures of the methylated ribonucleotides preserved after digestion (see Supplementary Figure S6).

It has also been reported that XRN1 plays a major role in rapid tRNA decay (RTD), which digests fully modified mature tRNA species *in vivo* (67,75–77). In one study, Whipple *et. al*. demonstrated the ability of XRN1 to digest through fully modified mature tRNAs using 3′ cytosine-3′,5′-bisphosphate (pCp) labelled tRNA^Ser(CGA)^ variants (77). The wild type tRNA^Ser(CGA)^ contained a 2′-*O*-methylated guanosine and uridine at the 18th and 44th ribonucleotide positions from 5′ end, respectively. When the tRNA variants were reacted with XRN1 the full length tRNA^Ser(CGA)^ was degraded to completion to give pCp as the end product. Additionally, the digestion of tRNA^Ser(CGA)^ and tRNA^Val(AAC)^ provided information on the ability of XRN1 to digest through other modified nucleotides such as *N^1^*-methylguanosine (m1G), ψ, dihydrouridine (D), m1A, 2,2,7-trimethylguanosine (m2,2,7G) and i6A (67,77). Thus, our single-molecule exosequencing strategy may possess the ability to identify these and other epitranscriptomic modifications without the need for antibody crosslinking or PCR amplification.

All eukaryotic mRNAs contain a cap structure at their 5′ end to enhance their stability by inhibiting the digestion ability of 5′-3′ exoribonucleases (78–81). The most common cap structure found in mRNA includes the 5′-5′ triphosphate linked 7-methylguanosine (m7G) cap, known as cap0, which is sometimes further modified by 2′-O-methylation at the first nucleotide, cap1 and second nucleotide, cap2 (78,81,82). Although less abundant, other cap structures such as nicotinamide adenine dinucleotide (NAD), m2,2,7G, and pyrophosphate groups are also found in mRNA (83–85). In our proposed exosequencing method, prior to XRN1 digestion the mRNA will need to be decapped to produce viable XRN1 substrates. We have successfully shown that RppH can remove the pyrophosphate group of IVT RNA products (see Supplementary Figure S3C). RppH can also be used for removing the cap0 structure of mRNA (85,86). In addition, Frindert *et al.* has demonstrated that *Bacillus subtilis* RppH (BsRppH), a homolog of RppH, can also be used to successfully remove the NAD cap structure *in vitro* resulting in 5′ monophosphorylated RNA for subsequent digestion by 5′-3′ exoribonucleases (83). Moreover, *Schizosaccharomyces pombe* Edc1-fused Dcp1–Dcp2 decapping enzyme, also known as ‘mRNA decapping enzyme (MDE)’, can be used to decap mRNA *in vitro* to remove both cap0 and cap1 structures (87). We successfully used MDE for decapping RNA prior to XRN1 digestion (see Supplementary Figure S4). The subsequent digestion of the decapped RNA by XRN1 further confirmed the ability of XRN1 to digest through 2′-*O*-methylated RNA as it was able to digest through the resulting 2′-*O*-methylated RNA following the decapping reaction (see Supplementary Figure S4C). UPLC analysis of the digestion products of the decapped 62mer RNA further confirmed successful decapping and digestion of 2′-*O*-methylated RNA (see Supplementary Figure S4D). In addition to removing cap0 and cap1 structures, MDE can also be used for decapping of m2,2,7G cap and also convert 5′ pyrophosphate ends to 5′ monophosphorylated ends (84,88). While the decapping of mRNA adds additional pre-processing steps, the decapping is simple using either RppH or MDE and requires only an incubation step followed by a quick RNA clean-up step for direct XRN1 RNA digestion.

In addition to using the solid-phase XRN1 reaction for single-molecule sequencing, solid-phase reactions can be used for IMER-based applications, which provide many advantages compared to solution-phase reactors, such as low sample consumption, high-throughput processing, and prevention of autodigestion of the enzyme (30,31,89). But, they also have limitations arising from poor enzyme kinetics due to mass transfer limitations (90,91). For a successful enzymatic reaction to occur, the substrate must diffuse to the immobilized enzyme. The use of a pillared IMER reduces this problem as it increases the surface-to-volume ratio compared to an open channel IMER as well as reducing diffusional distances (91). The IMER used in this study consisted of a microchannel with 3600 micropillars that were 100 μm in diameter and 60 μm in height with the inter-pillar spacing being 35 μm. The use of micropillars in this case increased the surface-to-volume ratio by 73% compared to an open channel IMER with the same dimensions. The higher digestion efficiency achieved in this report for the immobilized enzyme (87.6 ± 2.8%) compared to the free solution digestion (78.3 ± 4.4%; *P* = 0.0187) was possibly due to the higher surface area-to-volume ratio coupled with several other factors such as increased stability, and prevention of enzyme aggregation (31,92–94). The micropillared IMER also reduced diffusional distances increasing the number of interactions between the solution-borne RNA substrates and immobilized XRN1 enzymes. Furthermore, because single enzyme molecules are attached to the substrate most likely through a lysine residue that makes the active site accessible (see Figure 6), the RNA substrate is able to diffuse into the active site, whereas in free solution aggregation of enzyme molecules can make the active site inaccessible (31,95).

## CONCLUSIONS

In this report, we demonstrated for the first time the covalent attachment of XRN1 onto a solid-support for potential applications in single-molecule RNA exosequencing. The immobilization of XRN1 was carried out using established EDC/NHS coupling chemistry to a plastic support that was activated using UV/O_3_ light to create functional scaffolds containing –COOH groups. The immobilized XRN1 exhibited a digestion efficiency in terms of cleaving rate comparable to free solution XRN1 reactions indicating that surface immobilized XRN1 could be used in a RNA exosequencing approach. The ability of XRN1 to digest through methylated sequences, demonstrated in this report for both free solution and the immobilized enzyme using m6A and m5C methylated RNA sequences as models, is particularly advantageous for RNA sequencing, which could eliminate the need for antibodies and bisulfite treatment used in current NGS sequencing methods (66,96). The ability of XRN1 to cleave through these methylated residues was confirmed through data resulting from gel electrophoresis, fluorescence measurements, and UPLC/MS. Furthermore, we reported the clipping rate and the processivity of both free solution and immobilized XRN1. Immobilized XRN1 demonstrated a clipping rate of 26 ±5 nt s^−1^ and an apparent processivity of >10.5 kb. However, the clipping rate was secured at room temperature and not at 37°C, which is the optimal temperature for XRN1. However, the reported processivity is a lower limit for immobilized XRN1 because the number of nucleotides associated with the model (DMD) was completely digested. Therefore, it may be possible to read through the entire SARS-CoV-2 genome (∼30 kb) using our proposed exosequencing approach.

As noted, we are developing a single-molecule exosequencing approach, which uses a chip-based sequencer consisting of a solid-phase enzymatic bioreactor coupled to a column that measures in real time the electrophoretic mobility of free nucleotides with nanoscale electrophoresis following clipping using an exonuclease (19,21,22). While we have previously reported the use of λ-exonuclease solid-phase reactions for producing deoxynucleotide monophosphates, dNMPs (20), the work reported herein demonstrates the potential for repurposing the sequencer for RNAs. Previous work by our group has also shown that the dNMPs can be identified via their molecular-dependent electrophoretic mobilities at the single molecule level with a call accuracy >95% (21). Recently, we have also demonstrated that nanoscale electrophoresis can be used to identify the canonical rNMPs at a call accuracy >99% using a column made from a thermoplastic (97). Work is currently underway in our laboratory to couple the XRN1 solid-phase bioreactor with the nanoscale electrophoresis to provide a platform for single-molecule sequencing of RNA.

## Supplementary Material

gkab001_Supplemental_File
