# Supplementary material for: Solid-phase XRN1 reactions for RNA cleavage: application in single-molecule sequencing
Source: Nucleic Acids Res. 2021 Jan 28;49(7):e41. doi: 10.1093/nar/gkab001 (PMC8053086; doi:10.1093/nar/gkab001)
Supplement: gkab001_Supplemental_File [file gkab001_supplemental_file.docx]

**Supplemental Information**

**Solid-Phase XRN1 Reactions for RNA Cleavage: Application in Single-Molecule Sequencing**

Uditha S. Athapattu,**^1^** Charuni A. Amarasekara,^1^ Jacob R. Immel,^3^ Steven Bloom,^3^ Francis Barany,^4^ Aaron C. Nagel,^2*^ and Steven A. Soper^1,2,5,6 *^

^1^Department of Chemistry, University of Kansas, Lawrence, KS 66045, USA

^2^Sunflower Genomics, Inc., Lawrence, KS 66047, USA

^3^Department of Medicinal Chemistry, University of Kansas, Lawrence, KS 66045, USA

^4^Weill Cornell Medical College, New York, NY 10065, USA

^5^Department of Mechanical Engineering and Bioengineering, University of Kansas, Lawrence, KS 66045, USA

^6^Department of Cancer Biology and KU Cancer Center, University of Kansas Medical Center, Kansas City, KS 66160, USA

*Corresponding Authors – Steven A. Soper ([ssoper@ku.edu](mailto:ssoper@ku.edu)); Aaron C. Nagel ([aaron.nagel@sunflowergenomics.com](mailto:aaron.nagel@sunflowergenomics.com))

**Device fabrication and assembly.** The microfluidic devices used in this work were fabricated in poly(methyl methacrylate), PMMA (Plaskolite), using hot embossing. First, a brass mold master containing the required microstructures were micromilled into a brass plate using high precision micromachining (Kern, MMP Feinwerktechnik, Murnau-Westried, Germany). The microstructures on the brass mold master were then replicated into PMMA by hot embossing (155°C, 950 psi, 150 s) using a Precision Press model P3H-15-PLX (Wabash MPI, USA). After embossing, devices were diced using a bandsaw and reservoirs were drilled at each end of the microchannel. The devices were checked using non-contact profilometry (VK-X250, Keyence, Japan) to assess if the dimensions of the devices were consistent with the brass mold’s dimensions.

The microchannel of the pillared IMER was 24 mm long and 1.4 mm wide and contained 3,600 micropillars each of which were 100 μm in diameter and 60 μm in height. The pillar-to-pillar spacing of the device was 35 μm. The volume and the surface area of the device were 2.9 μL and 1.17 cm^2^, respectively. To allow for the real time monitoring of immobilized XRN1 digestion using fluorescence microscopy, a single channel microfluidic device was fabricated that consisted of a single flow channel (100 µm wide and 30 µm deep) made from PMMA and was hot embossed as noted above. Inlet and outlet reservoirs were drilled at each end of the microfluidic channel.


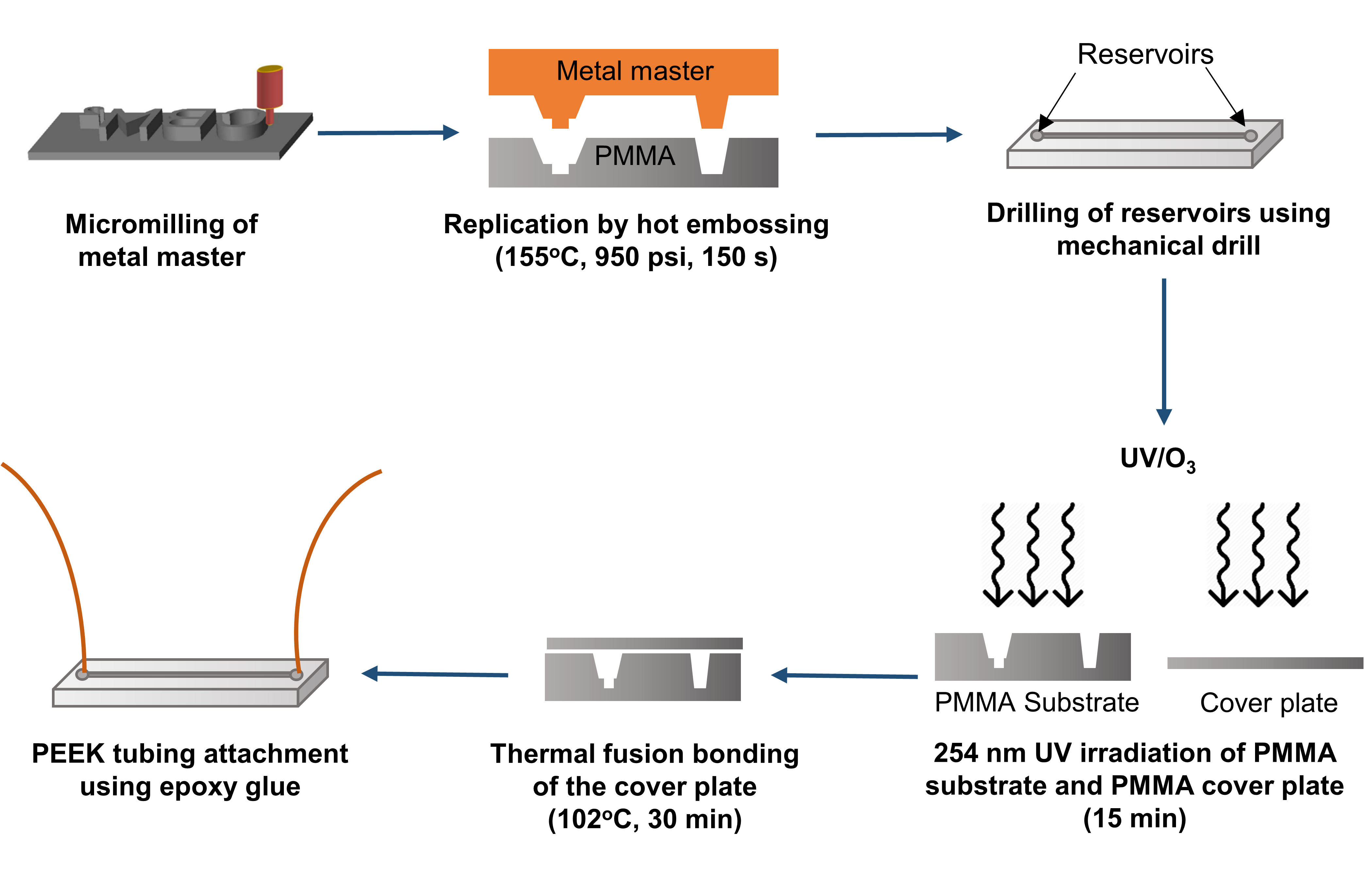
Before assembly, the microfluidic devices containing the embossed microstructures and cover plates (PMMA, 250 μm) were cleaned using isopropyl alcohol, 10% micro-90, and ddH_2_O followed by compressed air application to remove any debris from the microchannel. The cleaned devices were kept in an oven at 65°C for drying until further use.

**Figure S1.** Schematic representation of fabrication and assembly of microfluidic devices.

Prior to attachment of the cover plate to the embossed substrate, the devices and cover plates were UV/O_3_ irradiated at 254 nm (16 mW/ cm^2^) for 15 min, which created surface confined carboxylic acid groups to aid in the bonding as well as serving as a functional scaffold for the covalent attachment of the enzyme to the PMMA surface. After UV/O_3_ treatment, the cover plate was placed on top of the microfluidic device and both were clamped together between two borosilicate glass plates (McMaster, Atlanta, GA, USA) and then, inserted into a convection oven (ThermoFisher, USA), which was set at 102°C with the assembly remaining in this oven for 30 min. After thermal fusion bonding of the cover plate to the substrate, PEEK tubing (0.007–0.020″ i.d., 1/32″ o.d., Idex Health and Science) was attached to the reservoirs using epoxy glue to facilitate reagent/sample introduction into the microchannel. A complete schematic of the fabrication and assembly process is shown in Figure S1.

**Enzyme immobilization.** Covalent attachment of XRN1 to the photoactivated PMMA surface was carried out using EDC/NHS coupling chemistry for covalent immobilization of amine-containing biological material to surfaces containing –COOH groups (1,2). For the reaction, 200 mM EDC and 50 mM NHS in 0.1 M 2-[morpholino]ethanesulfonic acid (MES) buffer at pH 4.8 was introduced into the microfluidic devices and incubated at room temperature for 15 min. This enabled the formation of succinimidyl ester groups, which are labile to nucleophilic attack (3). When XRN1 was introduced into the microfluidic device, which displaced the EDC/NHS reagents, the amine groups on the enzyme reacted with the reactive succinimidyl ester groups forming an amide bond between the surface carboxyl groups and a primary amine resident within XRN1. After introduction of XRN1, devices were kept at room temperature for 2 h prior to washing away unbound enzyme with PBS and subsequently storing at 4°C until required for use.

**
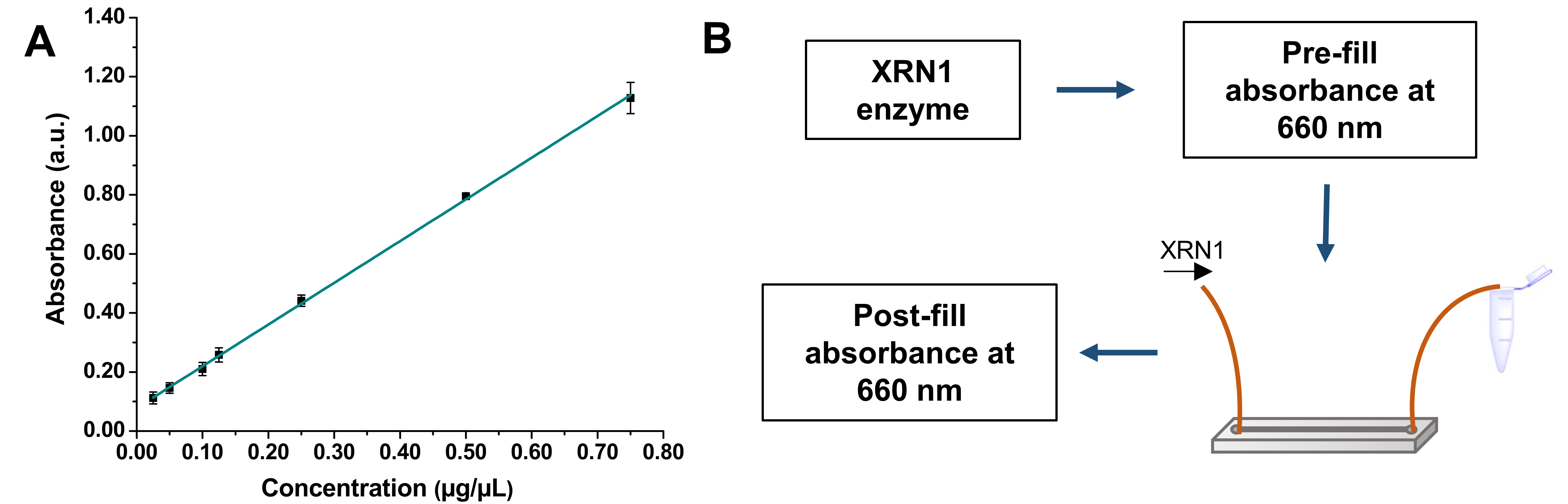
**Before introducing XRN1 to the EDC/NHS treated microfluidic devices, the storage buffer of the enzyme (20 mM Tris-HCl, 500 mM NaCl, 2 mM DTT, 0.1 mM EDTA, 50% Glycerol, 0.1% Triton X-100) was replaced with PBS. This was done because Tris-HCl in the storage buffer could interfere with the covalent attachment of the enzyme to the carboxylated plastic surface as Tris contains a primary amine group, which can compete with the enzyme for amide bond formation with the succinimidyl ester groups. Buffer exchange was carried out using 7 MWCO Zeba Spin columns (ThermoFisher Scientific, USA) according to the manufacturer’s protocol.

**Figure S2.** Quantification of immobilized enzyme on IMERs. **(A)** Calibration plot of Pierce 660 nm protein quantification assay (R^2^ = 0.9995). **(B)** Schematic representation of experimental procedure.

**Protein quantification.** For enzyme quantification, a Pierce 660 nm protein quantification assay (ThermoFisher Scientific, USA) was used according to the manufacturer’s directions. This assay uses a dye-metal based total protein quantification methodology and the complexation of the dye-metal complex onto a protein shifts the absorbance maximum to 660 nm from 450 nm (4). The calibration plot (R^2^ = 0.9995) for the assay plotted using BSA protein standards provided by the manufacturer is shown in Figure S2A.

The IMERs were assembled and prepared for enzyme immobilization as previously described. Absorbance of each XRN1 solution was measured at 660 nm before introducing 20 μL into the IMERs for enzyme covalent surface attachment. The eluant was collected and the absorbance was measured again at 660 nm. A schematic representation of the experimental procedure is shown in Figure S2B.

**Table S1.** Number of pmol of enzyme nonspecifically adsorbed.

| Concentration (nM) | Average pre-fill absorbance (n = 3) | Average post-fill absorbance (n = 3) | Adsorbed XRN1 amount (pmol) |
| --- | --- | --- | --- |
| 183 | 0.1323 ± 0.0007 | 0.1313 ± 0.0012 | 0.0142 ± 0.0086 |
| 305 | 0.1692 ± 0.0004 | 0.1689 ± 0.0002 | 0.0270 ± 0.0168 |
| 426 | 0.2061 ± 0.0002 | 0.2040 ± 0.0003 | 0.1672 ± 0.0247 |

| Concentration (nM) | Average pre-fill absorbance (n = 3) | Average post-fill absorbance (n = 3) | Immobilized XRN1 amount (pmol) |
| --- | --- | --- | --- |
| 183 | 0.1323 ± 0.0007 | 0.1036 ± 0.0083 | 2.32 ± 0.67 |
| 305 | 0.1692 ± 0.0004 | 0.1325 ± 0.0048 | 2.97 ± 0.38 |
| 426 | 0.2061 ± 0.0002 | 0.1558 ± 0.0082 | 4.07 ± 0.67 |

From the absorbance difference, the concentration difference was calculated. The number of moles of XRN1 that was lost due to immobilization was calculated using the concentration difference and the total input volume of XRN1. The negative control for this experiment consisted of introducing XRN1 solutions into the IMERs in the absence of EDC/NHS treatment. The number of moles of XRN1 that was nonspecifically adsorbed onto the surface was minimal as shown in Table S1. The number of moles of XRN1 lost to covalent immobilization onto the PMMA surface for each input solution is shown in Table S2.

**Table S2.** Number of pmol of enzyme lost to immobilization for each input concentration of XRN1.

**Plasmid DNA and PCR.** Plasmid Clone ID HsCD00082587 harboring the full-length dystrophin gene (DMD) open-reading frame was purchased from The PlasmID Repository within the [DNA Resource Core](http://dnaseq.med.harvard.edu/index.html) at [Harvard Medical School](http://hms.harvard.edu/). Briefly, a working stock of *Escherichia coli* DH5α was transferred to 50 mL of Luria Broth (LB) containing spectinomycin (100 µg/ mL) as the selective antibiotic marker and incubated overnight at 37°C in a rotary shaker. Plasmid DNA was isolated using mini-prep spin columns (Qiagen, MD, USA) following the manufacturer’s protocol, including the RNAse treatment step. Following isolation, purified plasmid DNA was quantified using a Biophotometer D30 (Eppendorf, NY, USA) to a final stock concentration of 100 ng/µL. Linearized FLUC DNA plasmid control template was obtained from New England Biolabs (NEB, Ipswich, MA, USA).

Full-length dystrophin cDNA was amplified from 50 pg plasmid pENTR223.1 DNA using a gradient PCR protocol in combination with the following primer pair for long-range, high-fidelity PCR using Hot Start LongAmp Mastermix (NEB, Ipswich, MA, USA): DMDR, 5’ – ATGCTTTGGTGGGAAGAAGTAGAG - 3’; DMDF/T7, 5’ - TGA GAC ACG GGC CAG AGC TGC CAG GAA ACA GCT ATG ACC ATG TAA TAC GAC TCA CTA TAG – 3’. DMDF/T7 contains a flanking T7 promoter sequence that is subsequently incorporated during PCR. The optimized PCR conditions were as follows: Initial denature at 94^o^C for 1 min followed by 28 cycles at 94^o^C for 15 s, 62^o^C for 10 s, 65^o^C for 50 s/kb. No final extension step was implemented to avoid addition of 5’/3’ dA overhangs following PCR that could potentially affect *in vitro* transcription reactions. The resulting PCR products were verified by standard gel electrophoresis on a 1% TBE agarose gel Bullseye Smartglow RNA/DNA stain (Midwest Scientific, Inc.; see Figure S3A for results). All lanes generated a discrete amplicon of approximately 11 kb and were subsequently pooled together for cleanup using a Monarch DNA/PCR Purification Kit (NEB, Ipswich, MA, USA) to generate DNA template that was used for *in vitro* transcription.

***
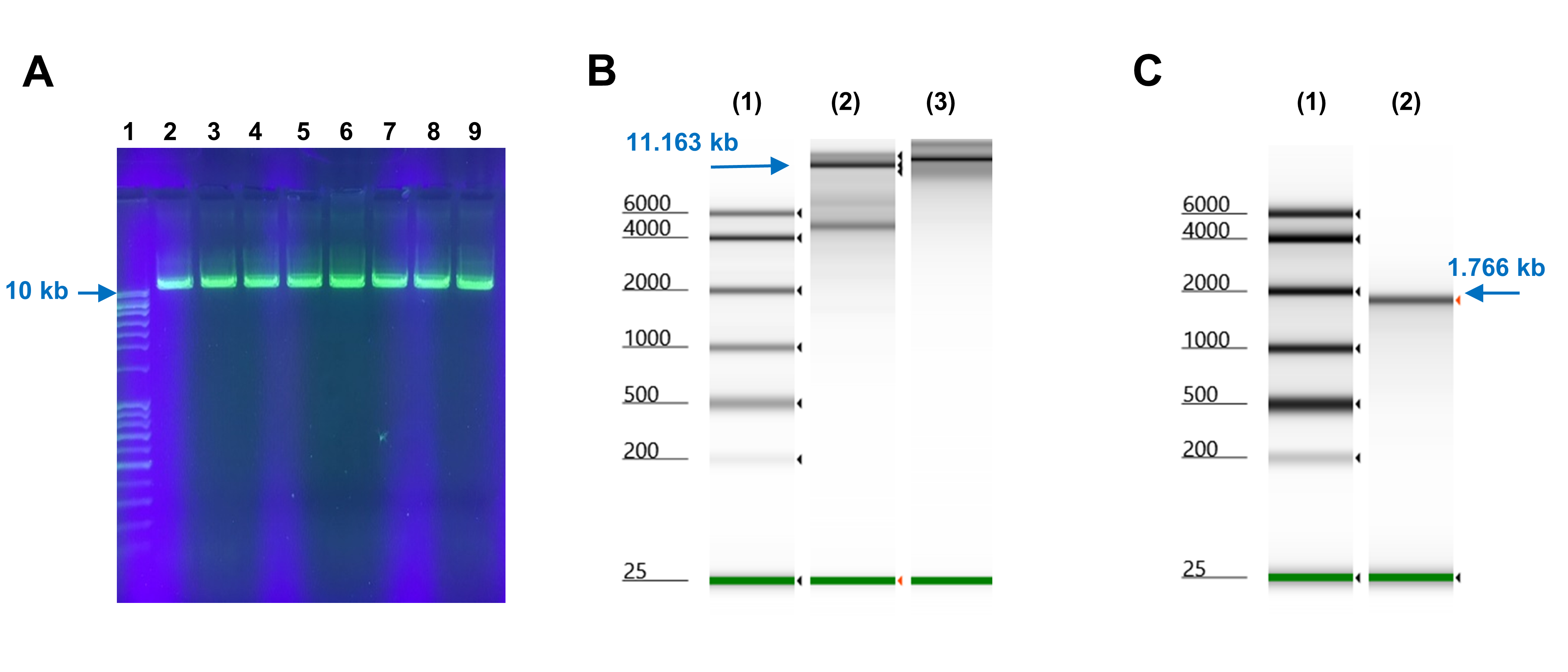
In vitro* transcription (IVT).** All IVT reactions were performed in a 20 µL final reaction volume using the HiScribe T7 High Yield RNA Synthesis kit (NEB, Ipswich, MA, USA) following the manufacturer’s recommended protocol. Following IVT, 10 µL of DNase cocktail containing 10 U of DNAse I (NEB, Ipswich, MA, USA), 3 µl 10X DNase I buffer, and 2 µL nuclease-free water was added to each IVT reaction to a final reaction volume of 30 µL and incubated for 20 min to degrade DNA template. Reactions were terminated by adding 5 µL of 50 mM EDTA solution, mixed, and briefly placed on ice. Synthesized RNA product was purified using a Monarch RNA Cleanup kit (NEB, Ipswich, MA, USA) following the manufacturer’s recommended protocol and eluted using nuclease-free water pre-warmed to 50°C to enhance recovery. Purified RNA product was quantified using a Biophotometer and diluted to a final stock concentration of 100 ng/µL in molecular-grade nuclease-free water (Midwest Scientific, Inc.).

**Figure S3.**  Gradient PCR amplification and IVT of full-length human dystrophin RNA (DMD) and FLUC RNA. **(A)** Agarose gel analysis of PCR product at the following gradient temperature profile: Lane **1** – DNA ladder; Lane **2** – 53.9^o^C; Lane **3** – 54.8^o^C; Lane **4** – 56.1^o^C; Lane **5** – 57.4^o^C; Lane **6** – 58.6^o^C; Lane **7** – 59.9^o^C; Lane **8** – 61.2^o^C; Lane **9** – 62.2^o^C. **(B)** High sensitivity RNA Tapestation analysis of DMD IVT products. **(1)** High sensitivity RNA ladder; **(2)** Purified IVT product. Blue arrow depicts the RNA band of interest, which was subsequently excised from an agarose gel; **(3)** Purified DMD RNA after gel excision, monophosphorylation, and purification. **(C)** High sensitivity RNA Tapestation analysis of FLUC IVT product. **(1)** High sensitivity RNA ladder; **(2)** Purified FLUC RNA after monophosphorylation and purification.

Initial evaluation of full-length RNA product was determined using a standard 1% non-denaturing TBE gel pre-stained with Bullseye Smartglow RNA/DNA stain (Midwest Scientific, Inc.). First, 10 µL of RNA product was mixed with 10 µL of 2X RNA loading dye (NEB, Ipswich, MA, USA) and heat denatured for 3 min at 72°C. Following denaturation, RNA samples were immediately loaded and resolved on a non-denaturing gel. IVT consistently resulted in an ~11 kb RNA transcript in addition to several additional co-synthesized RNA products. Several optimization strategies were employed to mitigate RNA co-synthesis such as time, temperature, and nucleotide concentration that still resulted in very similar banding patterns when compared to the recommended protocol. Because it was ideal for this work to use a homogeneous RNA species, we excised the corresponding 11 kb RNA band of interest from the non-denaturing agarose gel and used an RNA gel extraction and purification kit (Zymo Research, CA, USA). Results from the gel extraction consistently showed successful isolation and purification of non-degraded, full-length RNA product (see Figure S3B).

**Monophosphorylation of RNA transcripts**. The IVT RNA products that were synthesized according to the procedure outlined in the previous section were triphosphorylated at the 5’ end. Up to 500 ng of purified IVT RNA product was treated with RNA 5´ Pyrophosphohydrolase, RppH (NEB, Ipswich, MA, USA) to remove pyrophosphate from the 5´ end of the triphosphorylated RNA to generate 5´ monophosphate RNA following the manufacturer’s recommended protocol (see Figures S3B and S3C). Following incubation, RNA samples were pooled and purified using the Monarch RNA Cleanup kit (NEB, Ipswich, MA, USA) following the manufacturer’s recommended protocol and eluted in 50 µL nuclease-free water pre-warmed to 50°C to enhance recovery. Purified RNA product was quantified using a Biophotometer D30 and diluted to a final working stock concentration of 25 ng/ µL in molecular-grade nuclease-free water (Midwest Scientific, Inc.).

To demonstrate the ability to remove 5’ cap structures found in mRNA, we treated CleanCap Fluc RNA (TriLink Biotechnologies, San Diego, CA, USA) and IVT 62mer, both of which contained a cap1 structure at their 5’ end with mRNA decapping enzyme (MDE) prior to XRN1 digestion. The IVT 62mer was capped using “one-step capping and 2’-O-methylation protocol” that generates a cap1 structure at the 5’ end of the 62mer RNA following the manufacturer’s recommended protocol (see Figure S4A; NEB, Ipswich, MA, USA). The 62mer RNA had two extra guanosine groups at the 5’ end and the rest of the sequence was the same as the 60mer RNA (see Figure S5A). The capped RNAs were then treated with MDE to remove the 5’ cap1 structure (see Figure S4B). MDE belongs to the Nudix family of pyrophosphohydrolases, which reacts with polyphosphate groups to make monophosphorylated RNA (5-10). When MDE reacts with cap1 RNA, a m7G-pp- (m7GDP) group is removed and an intact RNA that is 2’-O-methylated (Nm) at the first nucleotide from the 5’ end is generated (see Figure S4B). MDE is reported as being highly efficient for removing both cap0 and cap1 structures (11).

Next, the decapped RNA samples were purified as previously described and reacted with XRN1. After digestion, the RNA samples were subjected to agarose gel electrophoresis (see Figure S4C). As can be seen from the gel images, the capped RNA remained intact when reacted with XRN1. When the RNA was decapped prior to XRN1 digestion. Loss of full-length intact RNA indicated that XRN1 was able to digest the RNA. Additionally, this experiment also demonstrated the ability of XRN1 to digest through the 2’-O-methylated RNA sequences as it was able to digest through the resulting 2’-O-methylated RNA sequences from the decapping reaction, which contained a 2’-O-methyladenosine (rAmMP) and 2’-O-methylguanine (rGmMP) for FLuc and 62mer, respectively.

For further confirmation, we analyzed the reaction products from the decapped 62mer RNA and XRN1 reaction using ultra-high-performance liquid chromatography (UPLC). UPLC was carried out using a Waters XBridge BEH C18 (2.5 μm, 4.6 x 150 mm) column and 100% (0.1% Formic acid/H_2_O) mobile phase with a 1.00 mL/ min flow rate. The reaction products contained the four canonical rNMPs, m7GDP and, rGmMP which confirmed successful decapping of the cap1-62mer RNA and subsequent digestion of the 2’-O-methylated RNA by XRN1 (Figure S4D).

**Figure S4.** Decapping of 5’ capped RNA. **(A)** Insertion of the 5’ cap1 structure to the IVT RNA using “one-step capping and 2’-O-methylation protocol”. **(B)** Cap1 removal using mRNA decapping enzyme (MDE) prior to XRN1 digestion. **(C)** Agarose gel analysis of capped and decapped RNA reactions with XRN1: lane **1,8** – RNA ladder; Lane **2-4** – Capped RNA without MDE and XRN1; Lane **5-7** – Caped RNA with XRN1; Lane **9-11** – Decapped RNA; Lane **12-14** – Decapped RNA with XRN1 **(D)** UPLC chromatograms of m7GDP standard solution and decapped 62mer digestion products. UV detection at 254 nm.


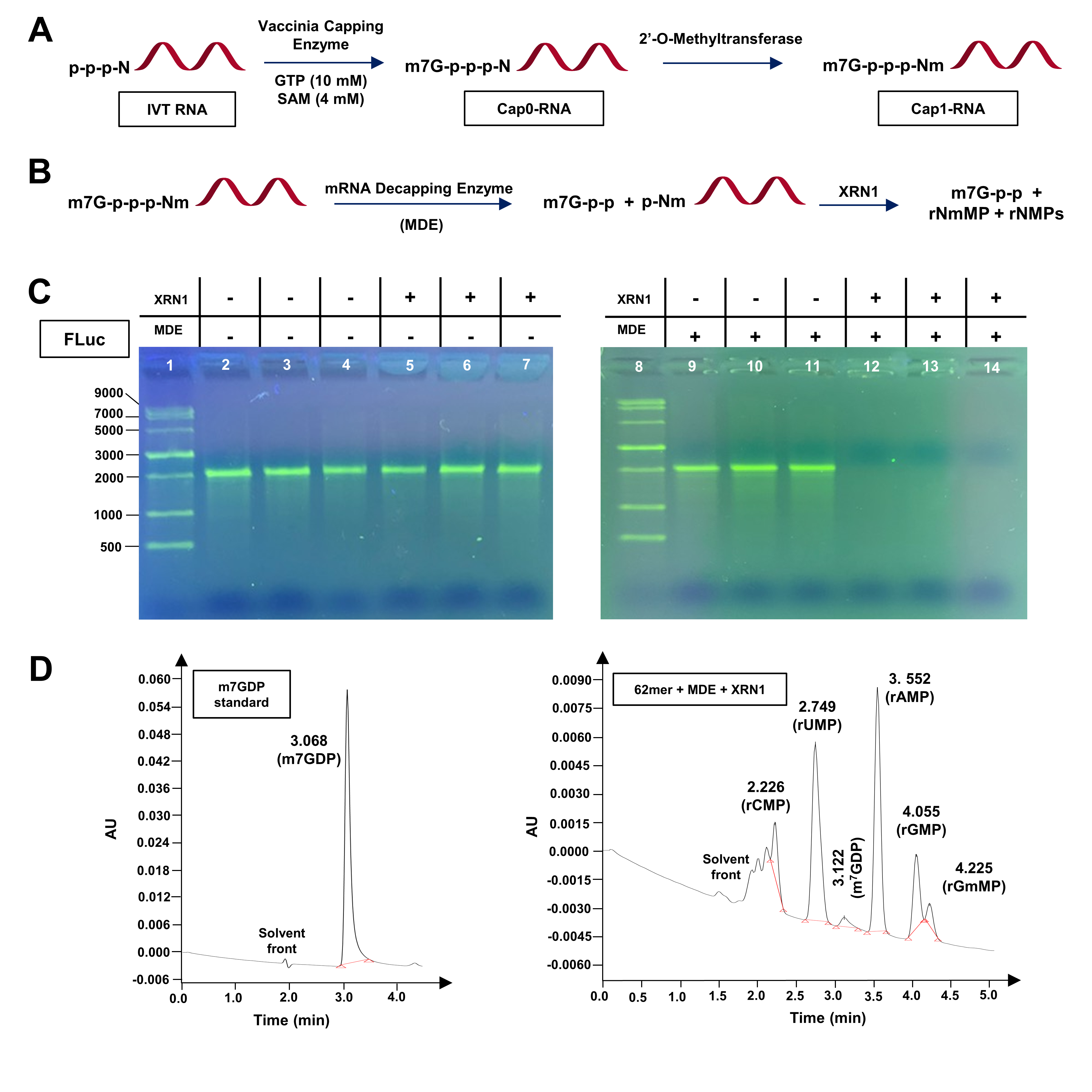


**Digestion of methylated RNA sequences.** The ability of XRN1 to digest sequences containing a methylated base was investigated using two sequences containing either an m6A (Figure S5A) or m5C (Figure S5B) at a specific nucleotide position near the oligomer’s 5’ end. A third unmethylated RNA strand (Figure S5C) was used as the control.

**Figure S5.** RNA sequences and digestion of methylated RNA. **(A)** Sequence of m6A methylated RNA. **(B)** Sequence of m5C methylated RNA. **(C)** Sequence of unmethylated control RNA. **(D)** Gel electrophoresis images for (1) RNA ladder, (2) 60 nt oligomer, (3) 56 nt oligomer, (4) 51 nt oligomer (5) 35 oligomer, and (6) mixture of 60, 51 and 35 nt oligomers. **(E)** Fluorescence emission spectra for m6A methylated RNA. **(F)** Fluorescence emission spectra for m5C methylated RNA.


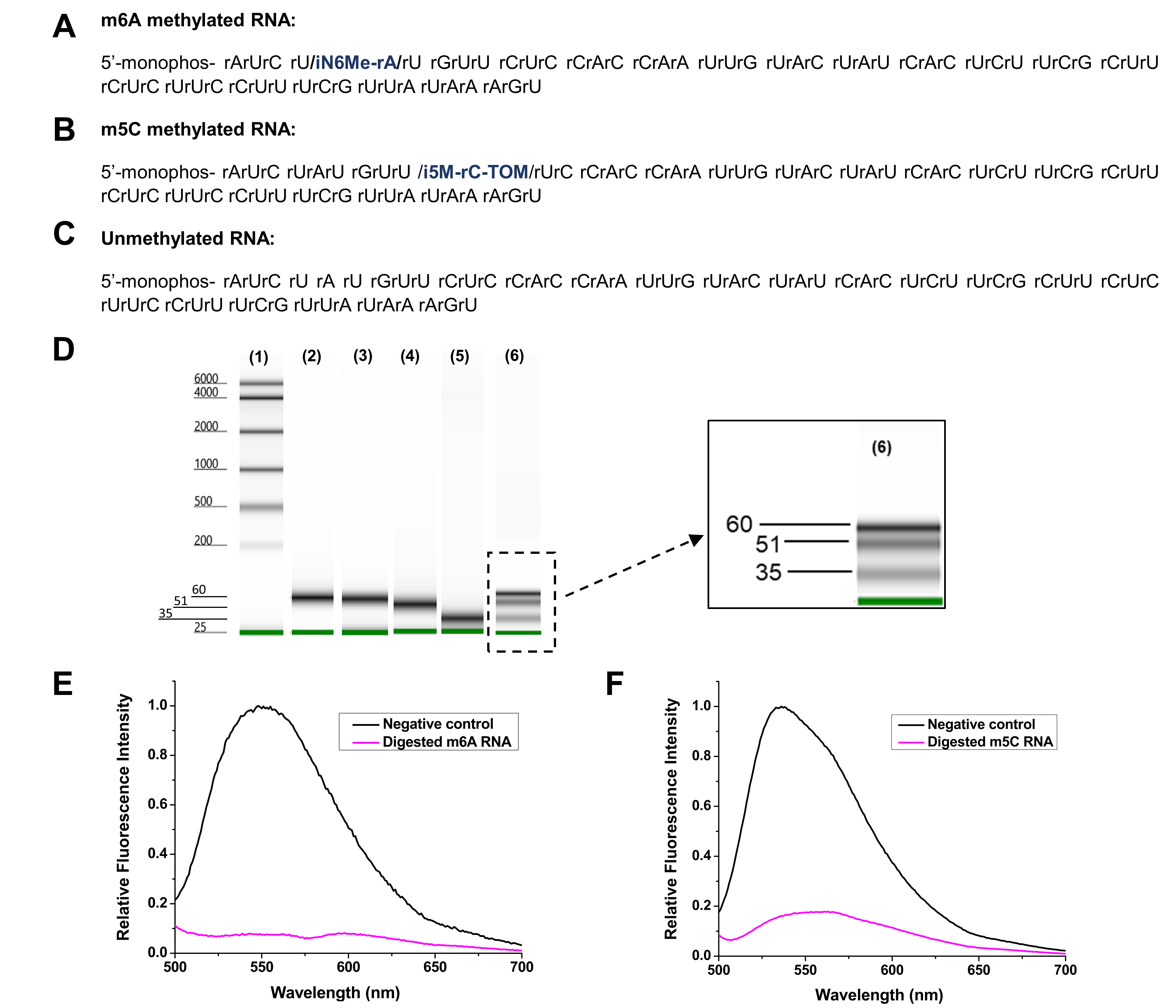


To determine the size of RNAs before and after digestion with XRN1, we used the Tapestation high sensitivity RNA Screentape gel electrophoresis assay following the manufacturer’s recommended protocol. The Tapestation high sensitivity RNA Screentape uses a microfluidic approach for gel electrophoresis. The Screentape, which is a non-rigid plastic device, contains 16 lanes of microfluidic channels each of which are 25 mm in length, 2 mm in width and 1 mm in height. The microchip gel electrophoresis is carried out separately in each lane and contains 3% *N-*acryloylamido ethoxyethanol (AAEE) as the gel sieving matrix. AAEE is reported in the literature to provide better performance than conventional poly(acrylamide) when used for nucleic acid separations (12). The gel also contains 3% of 2-[bis(2-Hydroxyethyl)amino]-2-(hydroxymethyl)propane-1,3-diol, which extends the useful electrophoresis life of the gel. The microchip gel electrophoresis can be considered as denaturing because the buffer used for the electrophoresis contains 50-75% DMSO, which will cause the RNA to be denature (13,14). The sample buffer also contains a staining dye, which is SYBR Gold that shows high binding affinity to nucleic acids. SYBR Gold is a highly sensitive dye with a quantum yield of 0.7 with a fluorescent enhancement of ~1000x upon binding to nucleic acids (15). A typical microchip gel electrophoresis reaction in the Tapestation is carried out at 24^0^C with an electric field of 100 V/cm. After separation, the band pattern is captured by a CCD camera in the instrument. The sensitivity of the RNA Screentape assay is stated to be 100 pg/ uL.


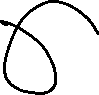


Due to the lack of data on the resolving power of the high sensitivity RNA Screentape assay, we carried out an experiment in which oligomers with lengths of 60, 56, 51 and, 35 were electrophoresed (see Figure S5D). The 60, 51, and 35 oligomers showed clear separation.

To determine the extent of digestion of both methylated RNA sequences by immobilized XRN1, the RNA solutions were collected after digestion for fluorometric analysis. The RNA samples were labelled with SYTO RNASelect Green dye and the fluorescence emission spectra were taken from 490 nm to 700 nm with 480 nm excitation. Peak area analysis of the emission spectra revealed that 87.0 ±4.2% (n = 4) and 77.3 ±6.0% (n = 3) of m6A (see Figure S5E) and m5C (see Figure S5F) methylated RNA were digested after 60 s, respectively, by the immobilized XRN1. Negative controls for each RNA consisted of passing RNA strands through devices with no immobilized XRN1.

**Analysis of XRN1 digestion products using ultra-high-performance liquid chromatography (UPLC) / Mass Spectrometry (MS).** To determine the identity of XRN1 reaction products using synthetic RNA oligomers as substrates (both modified and non-modified), a UPLC/MS analysis of the digestion products was performed. Before carrying out UPLC/MS analysis of the XRN1 digestion products, separation conditions were optimized using an rNMP mixture containing the canonical rNMPs and the modified rNMPs as well (m5C and m6A). The concentrations used for these experiments were based on their expected abundance within the synthetic RNAs (see Figure S6A).

Unmodified and modified 60 nt RNA strands were reacted with XRN1 and after the reaction was complete, XRN1 was removed using an Amicon Ultra 3K size exclusion column (Millipore Sigma, St. Louis, MO, USA) according to the manufacturer’s recommended protocol. UPLC/MS analyses of the digestion products were then performed using a Waters Acquity UPLC coupled to an Advion Expression^s^ CMS MS (electrospray ionization). UPLC used a Waters XBridge BEH C18 (2.5 μm, 4.6 x 150 mm) column and 100% (0.1% Formic acid/H_2_O) mobile phase with a 1.00 mL/ min flow rate.

As can be seen from the chromatograms (Figures S6B-S6D), the digestion products for all of the 60 nt RNA oligomers consisted of the rNMPs in their expected abundance based on the sequence content of the synthetic RNA oligomers. Mass spectra (M+H) analysis of the samples confirmed the presence of the rNMPs and that the methylations in the nucleotides were preserved following XRN1 digestion (see Figure S6E).


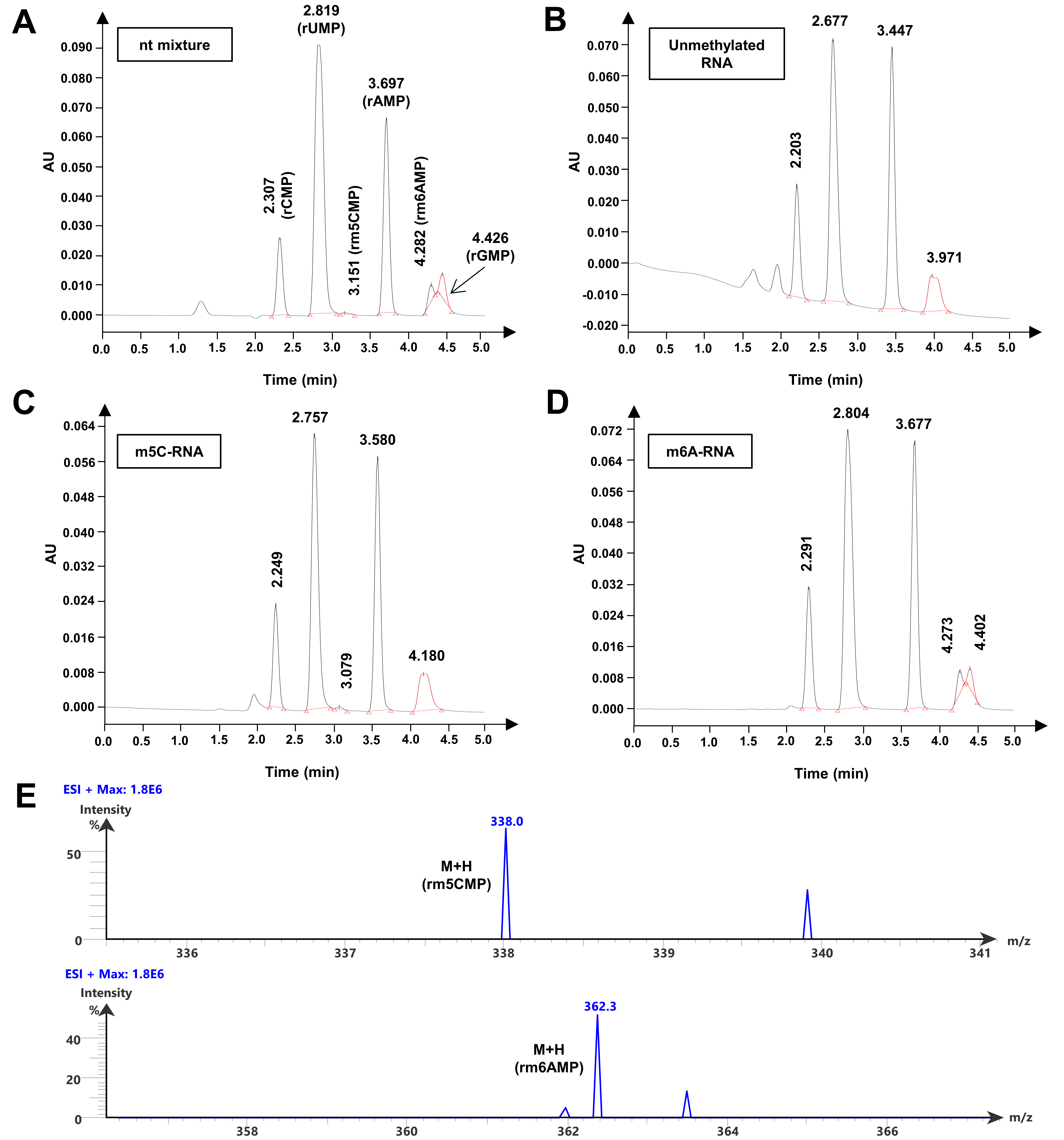
**RNA labeling and real time digestion measurements of RNA.** For real time fluorescence studies, FLUC RNA and DMD RNA were labelled either with SYTO 82 (541/560 nm; Life Technologies, Eugene, OR, USA) or RiboGreen (480/520 nm; Life Technologies, Eugene, OR, USA). SYTO 82 has an extinction coefficient >50,000 cm^-1^ M^-1^ and binds to both DNA and RNA exhibiting a quantum yield of 0.4, which is an approximate 40-fold enhancement compared to the unbound dye (16). RiboGreen, on the other hand, is specific to RNA and shows a fluorescence enhancement of 1,000 upon binding to RNA (17).

**Figure S6.** UPLC/MS analysis of digestion products from XRN1 reactions. Chromatograms (UV detection at 254 nm) of: **(A)** mixture of rNMPs; **(B)** unmethylated 60 nt synthetic RNA; **(C)** m5C methylated synthetic RNA; and **(D)** m6A methylated synthetic RNA. The UPLC/MS analysis was run after reaction with XRN1. **(E)** [M+H] peaks for m5C and m6A modified synthetic RNA oligomers obtained after digestion by XRN1.


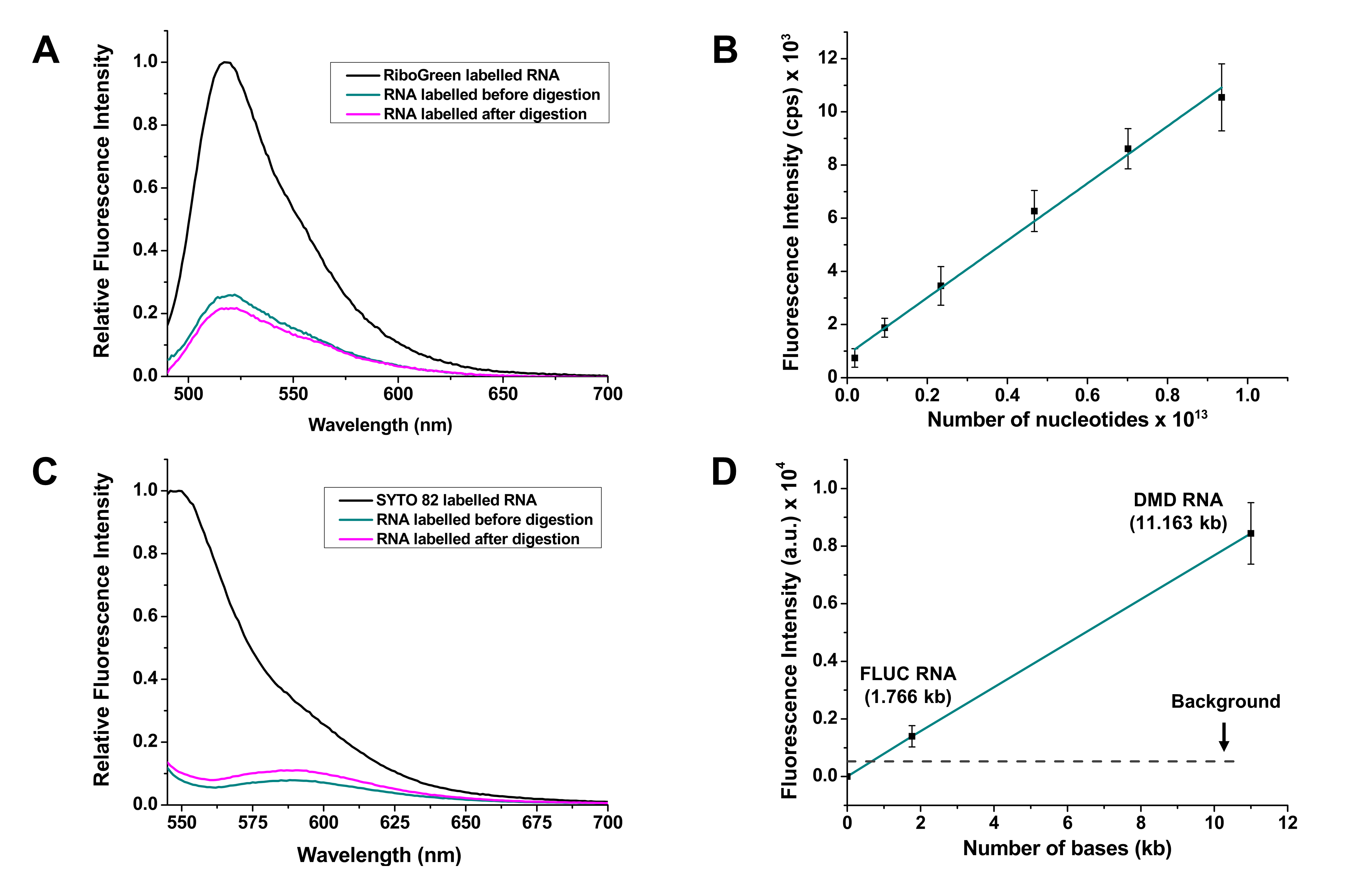
For labelling RNA with either of these dyes, a 5-fold molar excess of dye compared to the total number of nucleotides present in the strand was used. RNA solutions were heated at 72^o^C for 3 min and flash cooled in ice prior to adding the 5-fold molar excess of staining dye. The RNA-dye solutions were kept at room temperature for 30 min and the excess dye was removed from the solution using 7 MWCO size exclusion spin columns. Before using the stained RNA for experiments, 1X buffer was added with or without 10 mM MgCl_2_ (also included 100 mM NaCl, 50 mM Tris-HCl, and 1 mM DTT) according to the experimental need.

**Figure S7.** Effect of labelling on XRN1 activity and RNA calibration plots. **(A)** Fluorescence emission spectra of pre-labelled digestion and post-labelled digestion of RiboGreen labelled RNA. **(B)** Fluorescence intensity vs. number of nucleotides for RiboGreen labelled RNA (R^2^ = 0.9927). **(C)** Fluorescence emission spectra of pre-labelled digestion and post-labelled digestion of SYTO 82 labelled RNA. **(D)** RNA calibration plot for identification of the lowest detectable RNA fragment length using fluorescence microscope (R^2^ = 0.99996).

For determination of the clipping rate and processivity of both the free solution and immobilized XRN1 forms, dye-labelled RNA was used to monitor digestion in real time using fluorimetry or fluorescence microscopy. For the solution-phase clipping rate and processivity experiments, RiboGreen labelled FLUC RNA was used as the substrate. To determine the effect of RiboGreen labelling on the activity of XRN1, RiboGreen labelled and unlabeled RNA were reacted with XRN1 for 60 s and the fluorescence emission spectra of the solutions were taken. To assess the extent of digestion and compare the digestion percentage, peak area analysis was conducted. There was no statistical difference at the 95% confidence interval between pre-digestion labelling and post-digestion labelling using RiboGreen (see Figure S7A), which yielded a p value of 0.5196 (n = 4). To determine the number of nucleotides at each time interval, a calibration curve for the fluorescence intensity versus the number of nucleotides for RiboGreen labelled RNA was plotted (see Figure S7B). The number of nucleotides were calculated by multiplying the number of molecules of FLUC RNA at each concentration by the number of nucleotides in a FLUC RNA molecule (1,766 nucleotides).

For determining the immobilized enzyme’s clipping rate and processivity, real time monitoring of the SYTO 82 labelled DMD RNA digestion was undertaken. To determine the effect of SYTO 82 labelling on the activity of XRN1, labelled and unlabeled RNA were reacted with XRN1 for 60 s and the fluorescence emission spectra of the solutions were taken as was done for RiboGreen (see Figure S7C). Peak area analysis yielded a p value of 0.1573 (n = 3) indicated that labelling had no effect on activity. This suggested that the labelling conditions employed in this report did not inhibit the activity of XRN1. This agrees with previously reported work where it was shown that labelling with SYBR Green II dye (even after using 10X dye) did not have any impact on the activity of XRN1 (18).


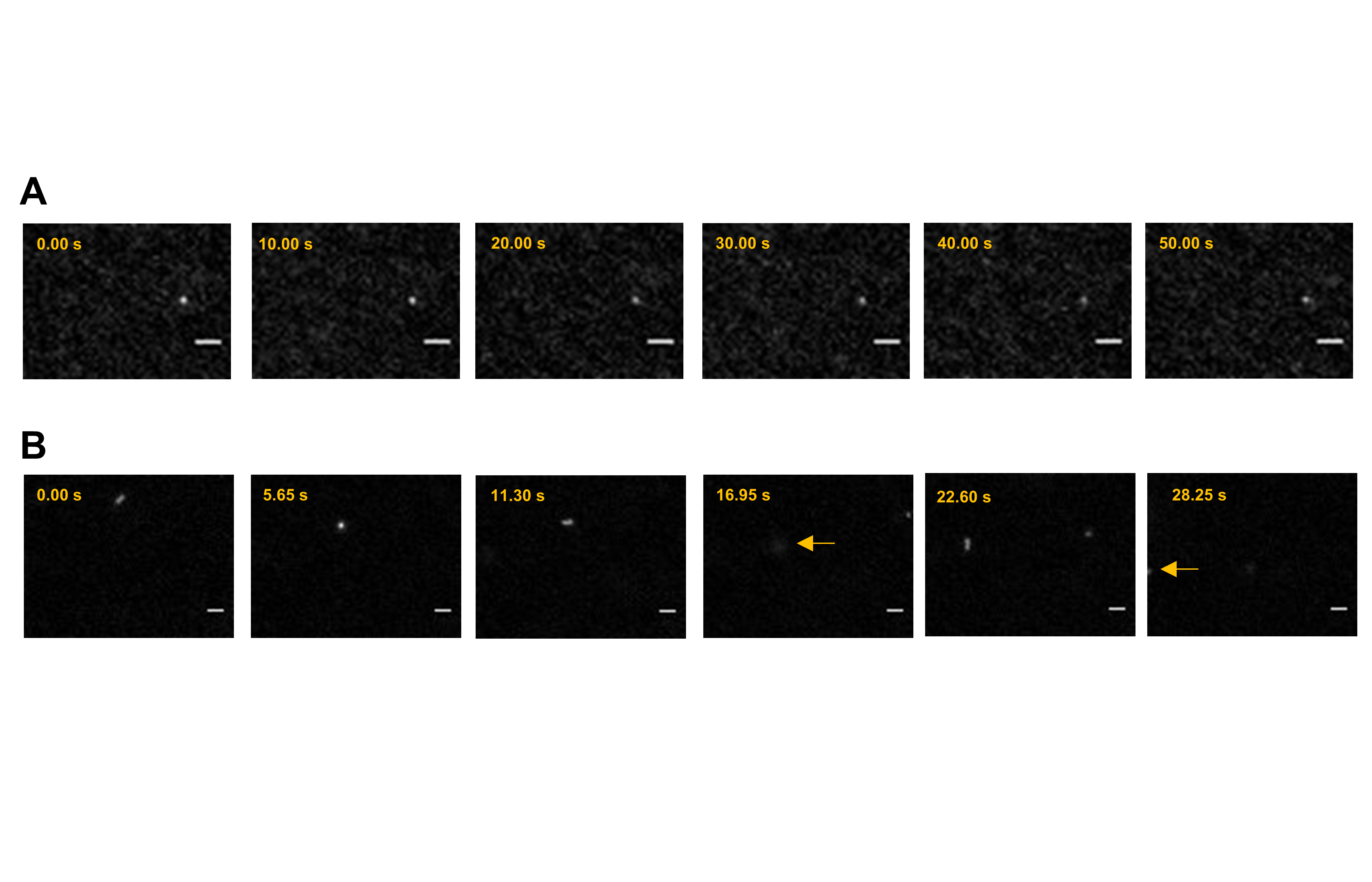
To determine the smallest detectable RNA fragment labeled with SYTO 82, a calibration plot was constructed using FLUC RNA and DMD RNA (see Figure S7D). According to the calibration plot, the smallest detectable SYTO 82 labelled RNA fragment distinguishable from the background was 664 nucleotides.

**Figure S8.**  SYTO 82 labelled DMD RNA in the single channel microfluidic device. **(A)** Labelled RNA-immobilized XRN1 complex on the cover plate of the microfluidic device. Due to complexation with immobilized XRN1, the RNA molecules remained stationary with time. **(B)** Free flowing labelled DMD RNA that is moving in and out of the imaging plane and eventually moving out of the field of view with time. The yellow arrow shows the position of the out-of-plane RNA molecule. The scale bar denotes 2 µm.

To determine the solid phase enzymatic clipping rate and processivity, it was important to distinguish between single molecules of RNA captured by the XRN1 enzyme immobilized to the cover plate surface of a microfluidic device from free-flowing RNA molecules. After immobilizing XRN1, dye-labeled RNA was introduced into the microfluidic device and was imaged through several imaging planes starting from the cover plate surface where the XRN1 enzyme was immobilized. Bright fluorescent spots were observed on the cover plate surface, which were found to be stationary even with fluid flow in the microfluidic channel and these spots were ascribed to RNA strands complexed with immobilized XRN1 (see Figure S8A). As imaging was done deeper into the microfluidic device, fluorescence spots indicative of free-flowing RNAs moving in and out of the field-of-view were observed (see Figure S8B), which we assumed arose from RNA not complexed to XRN1. This was verified with respect to the images shown in Figure S8A, where the RNA molecules in Figure S8B were moving and also, the fact that they disappeared whereas they did not in Figure S8A. Also, as noticed in Figure S8A, even after 50 s of constant
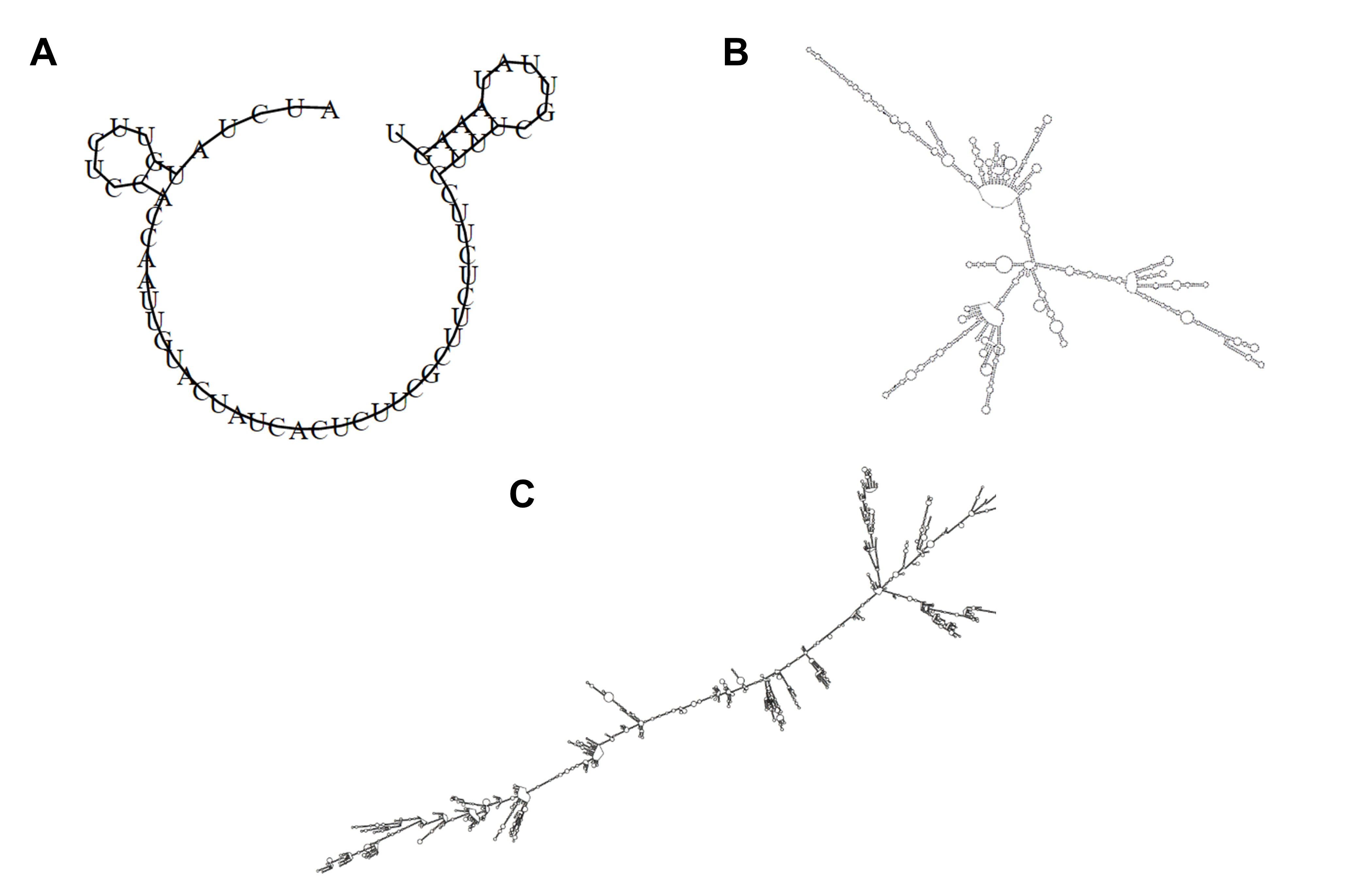
irradiation, photobleaching effects were minimal.

**Figure S9.** Minimum free energy (MFE) secondary structures of the **(A)** 60 b RNA, **(B)** FLUC RNA and **(C)** DMD RNA at room temperature. The MFE secondary structures were obtained using RNAfold webserver developed by Institute for Theoretical Chemistry, University of Vienna (8).

**RNA Secondary Structures.** All RNA substrates used in this report consisted of secondary structures at room temperature (25^0^C; see Figure S9). The Minimum Free Energy (MFE) secondary structures were obtained using RNAfold webserver developed by the Institute for Theoretical Chemistry at the University of Vienna (19). Due to the size restrictions of the server, only the first 10,000 nucleotides of the DMD RNA were input into the analyzer software. The MFEs of the most stable RNA secondary structures at room temperature were -1.99 kcal/mol for the 60 nt RNA, -574.76 kcal/mol for FLUC RNA, and -3581.78 kcal/mol for DMD RNA (10,000 nucleotides). If XRN1 was unable to digest through secondary structures, there would be partially digested RNA molecules remaining. As an example, the 60 nt RNA first stem-loop structure is encountered around the 5^th^ nucleotide from the 5’ end, which would leave an RNA fragment of ~55 nucleotides in length if the activity of XRN1 was stalled due to secondary structures. The fact that the 60 nt RNA digested to its end confirms the ability of XRN1 to digest through secondary structures.

**REFERENCES**

1. Jackson, J.M., Witek, M.A., Hupert, M.L., Brady, C., Pullagurla, S., Kamande, J., Aufforth, R.D., Tignanelli, C.J., Torphy, R.J. and Yeh, J.J. (2014) UV activation of polymeric high aspect ratio microstructures: ramifications in antibody surface loading for circulating tumor cell selection. *Lab on a Chip*, **14**, 106-117.

2. Wei, S., Vaidya, B., Patel, A.B., Soper, S.A. and McCarley, R.L. (2005) Photochemically patterned poly (methyl methacrylate) surfaces used in the fabrication of microanalytical devices. *The Journal of Physical Chemistry B*, **109**, 16988-16996.

3. Lim, C.Y., Owens, N.A., Wampler, R.D., Ying, Y., Granger, J.H., Porter, M.D., Takahashi, M. and Shimazu, K. (2014) Succinimidyl ester surface chemistry: implications of the competition between aminolysis and hydrolysis on covalent protein immobilization. *Langmuir*, **30**, 12868-12878.

4. Antharavally, B.S., Mallia, K.A., Rangaraj, P., Haney, P. and Bell, P.A. (2009) Quantitation of proteins using a dye–metal-based colorimetric protein assay. *Analytical biochemistry*, **385**, 342-345.

5. Decker, C.J. and Parker, R. (2002) mRNA decay enzymes: decappers conserved between yeast and mammals. *Proceedings of the National Academy of Sciences*, **99**, 12512-12514.

6. Deshmukh, M.V., Jones, B.N., Quang-Dang, D.-U., Flinders, J., Floor, S.N., Kim, C., Jemielity, J., Kalek, M., Darzynkiewicz, E. and Gross, J.D. (2008) mRNA decapping is promoted by an RNA-binding channel in Dcp2. *Molecular cell*, **29**, 324-336.

7. Grudzien‐Nogalska, E. and Kiledjian, M. (2017) New insights into decapping enzymes and selective mRNA decay. *Wiley Interdisciplinary Reviews: RNA*, **8**, e1379.

8. Li, Y. and Kiledjian, M. (2010) Regulation of mRNA decapping. *Wiley Interdisciplinary Reviews: RNA*, **1**, 253-265.

9. Mildvan, A., Xia, Z., Azurmendi, H., Saraswat, V., Legler, P., Massiah, M., Gabelli, S., Bianchet, M., Kang, L.-W. and Amzel, L. (2005) Structures and mechanisms of Nudix hydrolases. *Archives of biochemistry and biophysics*, **433**, 129-143.

10. Wulf, M.G., Buswell, J., Chan, S.-H., Dai, N., Marks, K., Martin, E.R., Tzertzinis, G., Whipple, J.M., Corrêa, I.R. and Schildkraut, I. (2019) The yeast scavenger decapping enzyme DcpS and its application for in vitro RNA recapping. *Scientific reports*, **9**, 1-9.

11. Paquette, D.R., Mugridge, J.S., Weinberg, D.E. and Gross, J.D. (2018) Application of a Schizosaccharomyces pombe Edc1-fused Dcp1–Dcp2 decapping enzyme for transcription start site mapping. *RNA*, **24**, 251-257.

12. Simò-Alfonso, E., Gelfi, C., Lucisano, M. and Righetti, P.G. (1996) Performance of a series of novel N-substituted acrylamides in capillary electrophoresis of DNA fragments. *Journal of Chromatography A*, **756**, 255-261.

13. Strauss Jr, J.H., Kelly, R.B. and Sinsheimer, R.L. (1968) Denaturation of RNA with dimethyl sulfoxide. *Biopolymers: Original Research on Biomolecules*, **6**, 793-807.

14. Brown, T., Mackey, K. and Du, T. (2004) Analysis of RNA by Northern and slot blot hybridization. *Current protocols in molecular biology*, **67**, 4.9. 1-4.9. 19.

15. Tuma, R.S., Beaudet, M.P., Jin, X., Jones, L.J., Cheung, C.-Y., Yue, S. and Singer, V.L. (1999) Characterization of SYBR Gold nucleic acid gel stain: a dye optimized for use with 300-nm ultraviolet transilluminators. *Analytical biochemistry*, **268**, 278-288.

16. Wlodkowic, D., Skommer, J. and Darzynkiewicz, Z. (2008) SYTO probes in the cytometry of tumor cell death. *Cytometry Part A: The Journal of the International Society for Analytical Cytology*, **73**, 496-507.

17. Jones, L.J., Yue, S.T., Cheung, C.-Y. and Singer, V.L. (1998) RNA quantitation by fluorescence-based solution assay: RiboGreen reagent characterization. *Analytical biochemistry*, **265**, 368-374.

18. Pellegrini, O., Mathy, N., Condon, C. and Bénard, L. (2008) In vitro assays of 5′ to 3′‐Exoribonuclease activity. *Methods in enzymology*, **448**, 167-183.

19. Lorenz, R., Bernhart, S.H., Zu Siederdissen, C.H., Tafer, H., Flamm, C., Stadler, P.F. and Hofacker, I.L. (2011) ViennaRNA Package 2.0. *Algorithms for molecular biology*, **6**, 26.
